# Near-Unity Nitrate to Ammonia conversion via reactant enrichment at the solid-liquid interface

**DOI:** 10.1038/s41467-025-60671-y

**Published:** 2025-07-01

**Authors:** Wanru Liao, Jun Wang, Yao Tan, Xin Zi, Changxu Liu, Qiyou Wang, Li Zhu, Cheng-Wei Kao, Ting-Shan Chan, Hongmei Li, Yali Zhang, Kang Liu, Chao Cai, Junwei Fu, Beidou Xi, Emiliano Cortés, Liyuan Chai, Min Liu

**Affiliations:** 1 Hunan Joint International Research Center for Carbon Dioxide Resource Utilization, State Key Laboratory of Powder Metallurgy, School of Physics, Central South University, Changsha, 410083 Hunan P. R. China; 2 School of Chemistry and Pharmaceutical Engineering, Changsha University of Science and Technology, Changsha, Hunan P. R. China; 3 Centre for Metamaterial Research & Innovation, Department of Engineering, University of Exeter, Exeter, UK; 4 Nanoinstitut München, Fakultät für Physik, Ludwig-Maximilians-Universität München, München, Germany; 5 National Synchrotron Radiation Research Center, Hsinchu, Taiwan; 6 Key Laboratory of Land Surface Pattern and Simulation, Institute of Geographic Sciences and Natural Resources Research, Chinese Academy of Sciences, Beijing, P. R. China; 7 State Key Laboratory of Environmental Criteria and Risk Assessment, Chinese Research Academy of Environmental Sciences, Beijing, P. R. China; 8 School of Metallurgy and Environment, Central South University, Changsha, P. R. China

**Keywords:** Heterogeneous catalysis, Energy, Electrocatalysis, Pollution remediation

## Abstract

Electroreduction of nitrate (NO_3_^‒^) to ammonia (NH_3_) is a promising approach for addressing energy challenges. However, the activity is limited by NO_3_^‒^ mass transfer, particularly at reduction potential, where an abundance of electrons on the cathode surface repels NO_3_^‒^ from the inner Helmholtz plane (IHP). This constraint becomes pronounced as NO_3_^‒^ concentration decreases, impeding practical applications in the conversion of NO_3_^‒^-to-NH_3_. Herein, we propose a generic strategy of catalyst bandstructure engineering for the enrichment of negatively charged ions through solid-liquid (S-L) junction-mediated charge rearrangement within IHP. Specifically, during NO_3_^‒^ reduction, the formation of S-L junction induces hole transfer from Ag-doped MoS_2_ (Ag-MoS_2_) to electrode/electrolyte interface, triggering abundant positive charges on the IHP to attract NO_3_^‒^. Thus, Ag-MoS_2_ exhibits a ~ 28.6-fold NO_3_^‒^ concentration in the IHP than the counterpart without junction, and achieves near-100% NH_3_ Faradaic efficiency with an NH_3_ yield rate of ~20 mg h^‒1^ cm^‒2^ under ultralow NO_3_^‒^ concentrations.

## Abstract (web summary)

Electroreduction of low-concentration NO_3_^−^ to NH_3_ is limited by NO_3_^−^ mass transfer. Here, the authors propose a strategy for NO_3_^−^ enrichment through charge rearrangement within the inner Helmholtz plane, achieving near-unity conversion of NO_3_^−^ to NH_3_.

## Introduction

Ammonia (NH_3_), one of the most common industrial chemicals, is crucial for the production of agricultural fertilizers and holds immense potential as a green hydrogen-rich fuel^1–3^. The current global ammonia demand exceeds 180 million tons annually, primarily fulfilled through industrial-scale production of energy-intensive Haber-Bosch (H-B) process^1,4^. Within this process, steam-reformed hydrogen (H_2_) undergoes reaction with nitrogen (N_2_) under elevated temperature (~ 500 °C) and pressure (> 100 atm)^5,6^, which not only accounts for approximately 1.4% of global carbon dioxide (CO_2_) emissions, but also necessitates the consumption of 2% of the world’s total energy supply^7,8^. Recently, electrocatalytic methodologies have surfaced as a viable clean energy pathway for decentralized ammonia synthesis at room temperature, accommodating a range of infrastructure scales and potentially powered by locally sourced renewable energy sources^9,10^. Despite the substantial global demand that may sustain the traditional ammonia production route in the near future, the electrochemical ammonia synthesis can be promising complementary process to the Haber–Bosch technology for contributing to decarbonizing ammonia production^11,12^.

Recently, diverse electrochemical approaches have been explored to address the varied demands for NH_3_ production in the future energy landscape, encompassing the electrochemical N_2_ reduction reaction (NRR)^13–15^, lithium-mediated NRR^16–19^ and nitrate reduction reaction (NO_3_RR)^20–22^. Among them, the present NH_3_ yield via NO_3_RR surpasses those of NRR by two to three orders of magnitude, mainly due to the relatively lower dissociation energy of the N=O bond (204 kJ mol^‒1^) of nitrate anion (NO_3_^‒^) compared to the N≡N bond (941 kJ mol^‒1^)^23,24^. Besides, NO_3_^‒^ exhibits a ubiquitous presence within contaminated groundwater and industrial effluent^25,26^, with their availability further augmented through the oxidation process of atmospheric N_2_ as the source of nitrogen^9,27,28^. Consequently, the NO_3_RR pathway has emerged as one of the most potential in renewable NH_3_ production.

While continuous progress has been made in NO_3_RR under high NO_3_^‒^ concentrations (> 100 mM)^11,29–31^, achieving large throughput and effective NO_3_^‒^ reduction in groundwater with concentrations below 10 mM remains a challenge, presenting a limited NH_3_ yield rate below 5 mg h^‒1^ cm^‒2^ and a Faradaic efficiency (FE) below 85%^22,32–34^. On the other hand, a considerable portion of the groundwater, stemming from both agricultural and industrial sources^35–40^, has a diluted concentration, necessitating a platform suitable for operation at low concentration. More importantly, the removal process of NO_3_^‒^ continuously reduces its concentration, inevitably reaching the low concentration regime with reduced performance.

To mitigate the degradation in low concentration, significant efforts have been devoted to concentrating NO_3_^‒^ locally around electrodes, employing various strategies such as porous carbon framework encapsulation^32^, built-in electric field regulation^41^, and nitrogen-vacancy engineering^42^. However, these endeavors primarily focus on manipulating catalyst properties for NO_3_^‒^ enrichment, with limited exploration of interfacial NO_3_^‒^ transfer under operational conditions. Particularly, at the reduction potential, an excess of electrons accumulates on the cathode surface, leading to a repulsion of negatively charged NO_3_^‒^ ions from the inner Helmholtz plane (IHP), where the catalytic reactions occur^27^. This ubiquitous effect of charge repulsion hinders the mass transfer and sets up an intrinsic bottleneck for the catalytic process.

**Fig. 1:**
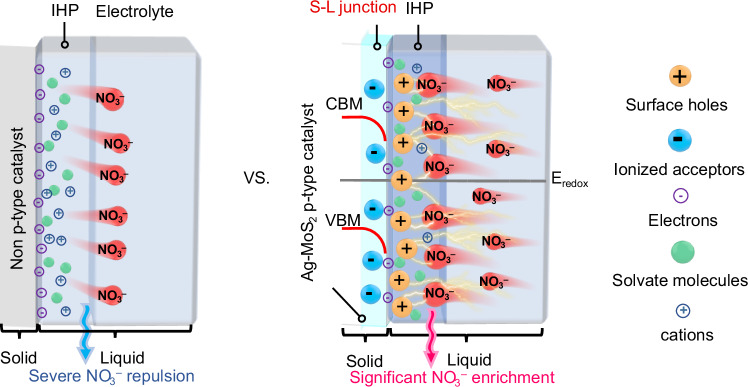
Solid-liquid (S-L) junction-mediated charge rearrangement to attract NO_3_^‒^. Schematic diagram of S-L junction-mediated NO_3_^‒^ enrichment within the IHP region over Ag-MoS_2_ at the reduction potential. *E*_redox_ represents the theoretical redox potential of NO_3_^‒^/NH_3_. CBM and VBM express the conduction band minimum and valence band maximum of Ag-MoS_2_, respectively. Note: IHP contains surface holes, solvate molecules, and NO_3_^‒^ anions and cations. Electrons accumulate on the electrode surface under negative polarization. Ionized acceptors exist in the S-L junction (see detailed discussions in the supplementary discussions).

Here, we overcome the fundamental limitation through utilizing a solid-liquid (S-L) junction^43,44^ to manipulate the charge distribution within the IHP region. By employing proper bandstructure engineering of the catalyst (*p*-type Ag-doped MoS_2_, Ag-MoS_2_), the enhanced interfacial charge transfer endows reinforced S-L junction to facilitate the NO_3_^‒^ enrichment in IHP (Fig. 1), showcasing a ~ 27.6-fold increase in NO_3_^‒^ concentration compared to the counterpart without junction. As a proof of concept, the Ag-MoS_2_ platform demonstrates impressive performance, including a record-high NH_3_ yield rate of ~ 20 mg h^‒1^ cm^‒2^ and NH_3_ FE of ~ 100%, outperforming previous results obtained under low NO_3_^‒^ levels^22,32,34,36^. Furthermore, the high NO_3_^‒^-to-NH_3_ conversion efficiency (99.3%) enables the removal of nitrate from 100 mM to low levels of 0.54 mM within 3 h, in sharp contrast with the reference (still relatively high level under 48 h operation). Notably, we successfully convert NO_3_^‒^ into high-purity NH_4_Cl products with near-unity efficiency by coupling the NO_3_RR with an air stripping process, demonstrating the potential of large throughput ammonia production in a sustainable way.

## Results

**Fig. 2:**
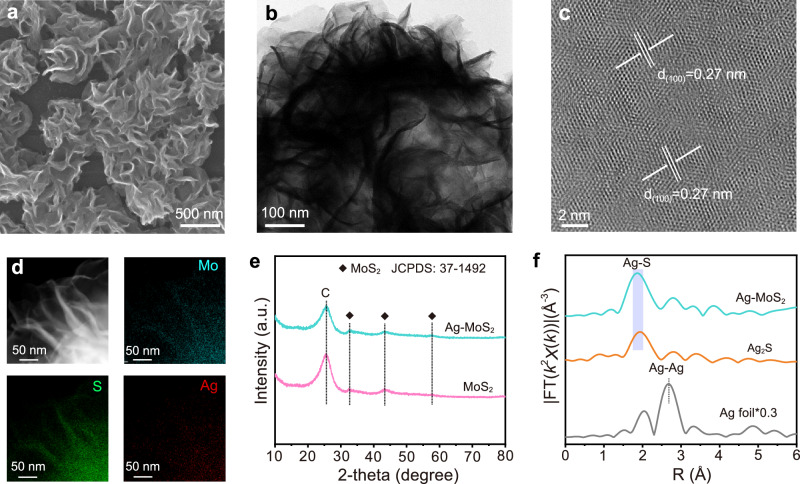
Synthesis and structural characterizations of Ag-MoS_2_. **a** SEM, (**b**) TEM, (**c**) HRTEM, and (**d**) EDX mapping of Ag-MoS_2_. **e** XRD patterns of Ag-MoS_2_ and MoS_2_. **f** Fourier transformed *k*^2^-weighted EXAFS spectra of Ag-MoS_2_ and reference samples.

Based on the merits of appropriate band gap (1.2–1.9 eV), excellent conductivity, adjustable Fermi level position, and two-dimensional characteristics of large specific surface area, MoS_2_ was selected as the platform for the electrocatalysis, with intentionally doped Ag as the enabler for the desired S-L junction formation. The Ag-doped MoS_2_ (Ag-MoS_2_) catalyst was grown in situ on a carbon cloth using a one-step hydrothermal method (Supplementary Fig. 1, see “Methods”). MoS_2_ as a reference was synthesized through a similar route, excluding the addition of a silver source. Scanning electron microscopy (SEM), transmission electron microscopy (TEM), and high-resolution TEM (HRTEM) revealed a nanoflower morphology of Ag-MoS_2_ with (100) crystal plane orientation (Fig. 2a–c and Supplementary Fig. 2), maintaining a morphology similar to that of MoS_2_ (Supplementary Figs. 3, 4). Energy-dispersive X-ray (EDX) elemental mapping illustrated the uniform distribution of Ag species on MoS_2_ nanoflowers (Fig. 2d). The X-ray diffraction (XRD) patterns of Ag-MoS_2_ and MoS_2_ were similar, indicating no formation of secondary phases after Ag doping (Fig. 2e). Inductively coupled plasma optical emission spectrometry (ICP-OES) determined the optimal Ag content in Ag-MoS_2_ to be 3.49 wt% (Supplementary Table 1).

To analyze the surface chemical states of the catalysts, high-resolution X-ray photoelectron spectroscopy (XPS) was performed (Supplementary Fig. 5). The Mo 3 *d* and S 2*p* XPS spectra indicated the presence of Mo^4+^ and S^2−^ in both Ag-MoS_2_ and MoS_2_^45,46^. In the Ag 3 *d* spectra, characteristic peaks at 368.16 and 374.13 eV were assigned to the Ag-S bond^47,48^. To further elucidate the coordination structure of Ag species, X-ray absorption fine structure (XAFS) was investigated. The Mo *K*-edge X-ray absorption near edge structure (XANES) spectra exhibited a higher pre-edge absorption energy of Ag-MoS_2_ than those of Mo foil and MoS_2_ (Supplementary Fig. 6a), implying that the doping Ag species induced decreased electron density on the Mo site. From the Ag *K*-edge XANES spectra, the absorption energy of Ag-MoS_2_ located between those of Ag foil and Ag_2_S references (Supplementary Fig. 6b), indicating the valence state of the doped Ag within 0 to 1. The Fourier-transformed extended XAFS (EXAFS) of Ag-MoS_2_ exhibited characteristic peaks of the Ag-S bond at 1.9 Å, confirming the coordination of Ag with S atoms (Fig. 2f). These comprehensive results provide clear evidence of the synthesis of Ag-doped MoS_2_ catalysts.

**Fig. 3:**
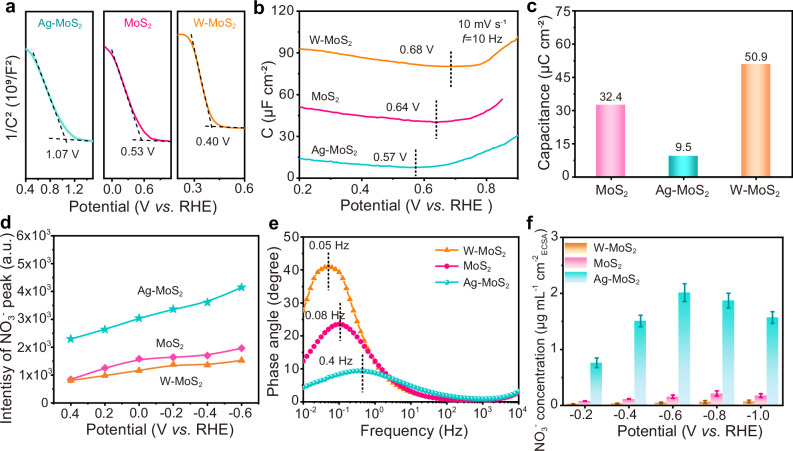
S-L junction-mediated NO_3_^‒^ enrichment within IHP region. **a** Mott-Schottky plots of Ag-MoS_2_, MoS_2_, and W-MoS_2_ catalysts. **b** The non-Faradaic capacitance-potential curves for the diverse catalysts. The potential of zero charge (PZC) describes the condition when the capacitance on a surface is minimal. **c** The fitted surface capacitance of the three catalysts. **d** The peak intensity of absorbed NO_3_^–^ from in situ Raman spectra of catalysts at various potentials. **e** Bode plots of catalysts at the reduction potential of − 0.6 V *versus* RHE in 10 mM NO_3_^–^ electrolyte. **f** The ECSA normalized NO_3_^‒^ adsorption capacity at different applied potentials on catalysts.

To investigate the construction and regulation of the solid-liquid (S-L) junction, we conducted ultraviolet photoelectron spectra (UPS) and Mott-Schottky (M-S) measurements. The UPS results (Supplementary Fig. 7) demonstrated a significant increase in the work function of Ag-MoS_2_ (5.47 eV) compared to MoS_2_ (4.98 eV), indicating a downward-shifted Fermi level after Ag doping. In addition to the UPS method, KPFM measurements were carried out to collaboratively demonstrative the increase of work function by Ag doping (Supplementary Figs. 8, 9)^49^. The surface potential difference between Ag-MoS_2_ and FTO substrate was ~ 230 mV, while 20 mV for MoS_2_. Correspondingly, the work functions of Ag-MoS_2_ and MoS_2_ were 5.13 and 4.92 eV, respectively. The variation trend was in consistent with UPS results, indicating a relatively high work function of Ag-MoS_2_. M-S plots further revealed the Fermi level potential (*E*_F_) of Ag-MoS_2_ (1.07 V *versus* RHE) and MoS_2_ (0.53 V *versus* RHE), respectively (Fig. 3a), surpassing the theoretical redox potential of NO_3_^–^/NH_3_ (*E*_redox_, 0.27 V *versus* RHE under neutral conditions (pH 6.71))^50–53^. The calculated carrier concentration of Ag-MoS_2_ is 1.14 × 10^19 ^cm^−3^, 1.8 times higher than that of MoS_2_ (6.3 × 10^18 ^cm^−3^, Supplementary Table 2). Thus, the Ag dopant as the electron acceptor to increase the carrier concentration and work function of MoS_2_ was verified.

Upon contact with the NO_3_^–^-containing electrolyte, the potential difference between the *E*_F_ and the *E*_redox_ (*E*_F_-*E*_redox_) triggered charge transfer between the catalyst surface and the inner Helmholtz plane (IHP), forming positively charged IHP on the electrolyte side (Supplementary Fig. 10). Specifically, p-type MoS_2_ catalysts exhibited the characteristic of hole conduction. Under the drive of *E*_F_-*E*_redox_, the holes (majority carrier) in the valance band would transfer from the semiconductor surface to the electrolyte, inducing charge rearrangement within the IHP region (Supplementary Fig. 10a)^54^. Meanwhile, the high carrier concentration (1.14 × 10^19 ^cm^−3^) and thin space charge layer width (5.88 nm) of Ag-MoS_2_ can contribute to the increase of probability for hole tunneling effect (Supplementary Fig. 10b and Supplementary Tables 2, 3), which benefited positive charges distribution within IHP. Compared to MoS_2_ with a limited *E*_F_-*E*_redox_ (0.23 V), the larger *E*_F_-*E*_redox_ value of Ag-MoS_2_ (0.76 V) provoked 0.53 V more downward band bending, intensifying the built-in electric fields at S-L junctions (see detailed quantitative methods and discussion in Supplementary Figs. 11–13).

**Fig. 4:**
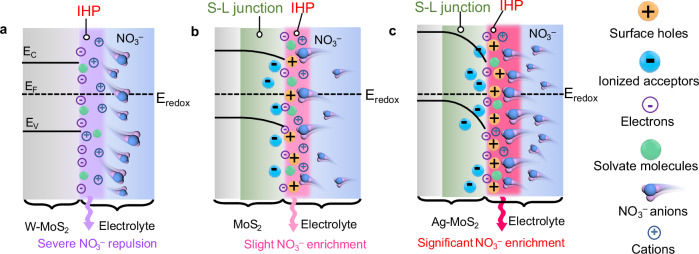
NO_3_^–^ distribution on IHP region over MoS_2_-based catalysts. **a** W-MoS_2_ without S-L junction, (**b**) MoS_2_ with weak S-L junction, and (**c**) Ag-MoS_2_ with strong S-L junction at the reduction potential.

To explore the influence of S-L junction on the charge distribution within the IHP region, alternating current voltammetry (ACV) measurements were employed (Fig. 3b)^55,56^. ACV was a non-destructive technique which had been widely utilized to record the adsorption behavior change in the IHP range^57,58^. Compared to MoS_2_, Ag-MoS_2_ exhibited a negatively shifted potential of zero charges (PZC, 0.57 V *versus* RHE). PZC was adopted as an indicator for the IHP structure evolution, and the negative PZC indicated the adsorption of anion on the IHP region of Ag-MoS_2_ (Fig. 4 and Supplementary Figs. 14, 15)^59,60^. To investigate the charge distribution under reduction potential, surface capacitance (charge distribution within the solid-liquid interface) of the catalysts was obtained from Nyquist plots at − 0.6 V *versus* RHE (Supplementary Fig. 16). The capacitance of Ag-MoS_2_ (9.5 μC cm^−2^) was lower than that of MoS_2_ (32.4 μC cm^−2^), indicating significant positive charges distribution of Ag-MoS_2_ (Fig. 3c). High valence metals had been reported to serve as electron donors to regulate the *E*_F_ away from VBM, for enhancing the n-type characteristics of semiconductors^61^. Thus, we also prepared the reference samples with tungsten (W) doping, to demonstrate the effect of the S-L junction. In contrast, a n-type W-doped MoS_2_ (W-MoS_2_) counterpart with no obvious S-L junction (Fig. 3a and Supplementary Figs. 7 and 17–21) had higher PZC value (0.67 V *versus* RHE, Fig. 3b) and capacitance (50.9 μC cm^−2^, Fig. 3c), suggesting extensive anions packed on its IHP. These results confirmed that Ag doping can effectively enhance the built-in electric field at the S-L junction, which consequently induced abundant positive charges distributed on the IHP of Ag-MoS_2_ at the reduction potential.

To investigate the S-L junction-mediated NO_3_^‒^ enrichment effect, in situ Raman spectra of the three catalysts were carried out (Supplementary Fig. 22). Apart from the observed H_2_O peak (at 1605 cm^−1^)^62–64^, the NO_3_^‒^ peak (at 1046 cm^−1^)^65,66^ was detected at the potentials from open circuit potential (OCP) to − 0.6 V *versus* RHE, indicating NO_3_^‒^ adsorption near the catalyst surface. Notably, Ag-MoS_2_ with a stronger NO_3_^‒^ peak than those of MoS_2_ and W-MoS_2_ at each reduction potential (Fig. 3d) demonstrated a pronounced NO_3_^‒^ enrichment effect. In Bode phase plots (Fig. 3e), compared with W-MoS_2_ (0.05 Hz) and MoS_2_ (0.08 Hz), the higher frequency of Ag-MoS_2_ (0.4 Hz) implied a facilitated interfacial charge transfer process, which can be ascribed to its intensified NO_3_^‒^ accumulation within the IHP region^67,68^. Elemental mapping displayed obvious N and O signals on Ag-MoS_2_ nanoflowers during the electrolysis of −0.6 V *versus* RHE in NO_3_^‒^-contained electrolyte, whereas they were relatively shallow or inconspicuous on MoS_2_ and W-MoS_2_ (Supplementary Figs. 23–25), disclosing a large number of NO_3_^‒^ migrated to the surface.

To quantify the NO_3_^‒^ concentration, the NO_3_^‒^ enrichment capacity of the three catalysts was measured by ion chromatography (IC) at applied potentials (Fig. 3f and Supplementary Fig. 26, see “Methods”^69^). By normalizing the NO_3_^‒^ adsorption capacity with respect to the electrochemical active surface area (ECSA), the concentration of NO_3_^–^ adsorbed on Ag-MoS_2_ presented a normal distribution trend as the potential ranging from − 0.2 to − 1.0 V *versus* RHE (Fig. 3f and Supplementary Fig. 27), and reached the peak at − 0.6 V *versus* RHE (2.0 μg mL^‒1^ cm^‒2^_ECSA_). Due to the different Fermi level position, the adsorbed NO_3_^–^ concentration of MoS_2_ and W-MoS_2_ peaked at more negative potentials of − 0.8 and − 1.0 V *versus* RHE, respectively. As the Ag dopant acted as the electron acceptor to increase the carrier concentration (Supplementary Table 2), it induced an intensified surface band bending when the Ag-MoS_2_ was contacted with the NO_3_^–^ contained electrolyte. Thus, the maximum adsorbed NO_3_^–^ concentration of Ag-MoS_2_ surpassed MoS_2_ and W-MoS_2_ by factors of 8.8 and 27.6, respectively, at their optimal adsorption potentials. The NO_3_^‒^ adsorption concentration was also normalized by specific surface area (SSA), this trend aligned with the ECSA normalization results (Supplementary Fig. 28), highlighting superior intrinsic NO_3_^‒^ adsorption capability of Ag-MoS_2_. The lowest surface capacitance at −0.6 V *versus* RHE suggested massive positive charges within IHP (Supplementary Fig. 29), contributing to the peaked NO_3_^‒^ concentration at that potential. While the excessive potential (above −0.6 V) triggered severe downward shift of *E*_F_ into the valence band of Ag-MoS_2_ (Supplementary Fig. 30 and Supplementary Table 4, see details in the corresponding discussion), forming a degenerate semiconductor with metallic property, as proved by the decreased arc radius at high-frequency region. This could severely destroy the S-L junction to impact the enrichment of NO_3_^‒^. We also implemented finite element method simulations for the NO_3_^‒^ distribution on the three catalyst surfaces through COMSOL, which matched the experimental results demonstrated (Supplementary Fig. 31 and Supplementary Table 5).

Under the S-L junction-induced NO_3_^‒^ enrichment feedback, we evaluated the NO_3_RR performance in a standard three-electrode system at ambient temperature and pressure. In this reaction system, NH_4_^+^, NO_3_^‒^, and NO_2_^‒^ were monitored and quantified by coloration and ^1^H nuclear magnetic resonance (NMR) spectroscopy^70^ (Supplementary Figs. 32–35). A typical industrial and agricultural groundwater-relevant NO_3_^‒^ concentration of 10 mM was reasonably used in the electrolyte for the standard electrochemical tests^35,37,38,69^. Linear sweep voltammetry (Supplementary Fig. 36) curves of W-MoS_2_, MoS_2_, and Ag-MoS_2_ all presented a higher current density between −0.5 and − 1.65 V *versus* RHE in the NO_3_^‒^ electrolyte relative to NO_3_^‒^-free solutions. Particularly, the most significant current difference occurred on Ag-MoS_2_, revealing its promising NO_3_RR activity.

**Fig. 5:**
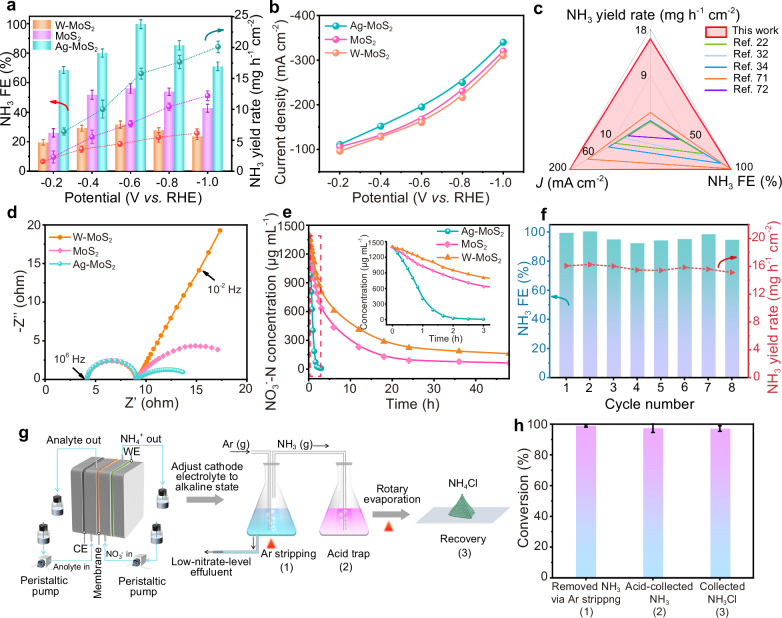
NO_3_RR performance under ultralow NO_3_^‒^ concentrations. **a** NH_3_ yield rate and NH_3_ FE of catalysts in 10 mM NO_3_^–^ electrolyte at various applied potentials. **b** *I*–*V* plots of catalysts in 0.5 M K_2_SO_4_ with 10 mM NO_3_^‒^ electrolyte at various potentials for 0.5 h electrolysis. **c** A summary of recent NO_3_RR works on NH_3_ yield rate, NH_3_ FE and current density under ultralow NO_3_^‒^ concentrations (NO_3_^‒^ concentration ≤ 10 mM)^22,32,34,71,72^. **d** EIS tested at −0.6 V *versus* RHE in 10 mM NO_3_^–^ electrolyte. **e** NO_3_^–^ removal in initial 0.5 M K_2_SO_4_ with 100 mM NO_3_^–^ electrolyte at − 0.6 V *versus* RHE in H-cell reactor. After 3 h of electrolysis, only 0.54 mM  of NO_3_^–^–N. Insert was the enlarged vision within 3 h. **f** NO_3_RR performance stability over Ag-MoS_2_ measured in a 0.5 M K_2_SO_4_ with 10 mM NO_3_^–^ electrolyte at − 0.6 V *versus* RHE. **g** Schematic of the ammonia product synthesis process from 100 mM NO_3_^–^ electrolyte to NH_4_Cl at − 1.0 V *versus* RHE. **h** The conversion efficiency of different steps for the ammonia product synthesis process. Numbers on the *x*-axis indicated the corresponding conversion steps in panel (g). Error bars indicate the relative standard deviations of the mean (*n* = 3).

Following the chronoamperometry measurements at various applied potentials tested in a flow cell reactor (Supplementary Fig. 37), the NO_3_RR performance was assessed for the three catalysts with a 0.5 h duration. Of these, Ag-MoS_2_ displayed the superior performance (Fig. 5a, b), with a near unit NH_3_ Faradaic efficiency (FE) of 99.7% at − 0.6 V *versus* RHE (~ 200 mA cm^−2^) and an optimal NH_3_ yield rate of 20.1 mg h^‒1^ cm^‒2^ at − 1.0 V *versus* RHE (~ 340 mA cm^−2^). The optimal NH_3_ yield rate value was about 3.4-folds and 1.7-folds than those of W-MoS_2_ and MoS_2_, respectively. Similarly, the peak NH_3_ FE of Ag-MoS_2_ was approximately 3.2-folds and 1.8-folds than those of W-MoS_2_ and MoS_2_, respectively. These results displayed the superior NO_3_RR performance of Ag-MoS_2_ compared to its counterparts. Moreover, the performance surpassed most state-of-the-art NH_3_ activity ever reported at a low NO_3_^‒^ system (Fig. 5c and Supplementary Table 6)^22,32,34,71,72^. The electrochemically active surface area-normalized NH_3_ yield rate also clarified the excellent intrinsic activity of Ag-MoS_2_ (Supplementary Figs. 27 and 38). The blank experiments without adding NO_3_^‒^ to the electrolyte or working at OCP produced a negligible amount of NH_3_ (Supplementary Fig. 39), excluding the possible interference on the quantification results. Besides, the possible contribution of Ag nanoparticles on the NO_3_^‒^ enrichment and NO_3_RR performance of MoS_2_ has been eliminated (Supplementary Figs. 40–43).

To reveal the significance of NO_3_^‒^ enrichment for superior NO_3_RR performance under a low-NO_3_^‒^ concentration system, EIS measurements were conducted (Fig. 5d and Supplementary Fig. 16). In the Nyquist plots, semicircles with similar radii at the high-frequency region of the three samples indicated nearly equivalent conductivity. At the low-frequency region, W-MoS_2_ without S-L junction showed a straight line with a slope of 45°, suggesting that NO_3_^‒^ mass transfer was the rate control step for NO_3_RR. In contrast, the smaller arc radius over Ag-MoS_2_ illustrated the enhanced reaction rate of NO_3_RR (from 16.41 to 6.92 Ω cm^‒2^, Supplementary Table 7). The lowest phase angle of Ag-MoS_2_ in bode plots also testified its accelerated catalytic kinetics, ascribing to the distinct NO_3_^‒^ enrichment effect (Fig. 3e). In addition, from the NO_3_RR performance at a wide range of NO_3_^‒^ concentrations (Supplementary Fig. 44), the optimal Ag-MoS_2_ in K_2_SO_4_ system (Supplementary Figs. 45–46) exhibited its broad adaptability and achieved an excellent NH_3_ FE at − 0.6 V *versus* RHE (> 90%, in Supplementary Fig. 47), even at ultra-low concentration (1 mM). While the NH_3_ yield rate of Ag-MoS_2_ was significantly higher than those of W-MoS_2_ and MoS_2_ in low NO_3_^‒^ concentration (Supplementary Fig. 44, insert), it gradually became comparable as the concentration increased. These results highlighted the importance of NO_3_^‒^ enrichment to overcome the constraint of low-concentration NO_3_^‒^ electroreduction to NH_3_, via the S-L junction-mediated effect.

To examine the NO_3_^‒^ removal capability of Ag-MoS_2_, the conversion tests with an initial 100 mM NO_3_^‒^ were carried out in an H-cell reactor, and the remaining products were measured. Impressively, the NO_3_RR on the three samples followed typical quasi-first-order kinetics, and the NO_3_^‒^ to NH_3_ selectivity of Ag-MoS_2_ was up to 99.3% within only 3 h of electrolysis at − 0.6 V versus RHE (NO_3_^‒^ concentration decreased sharply from 100 mM to 0.54 mM, Fig. 5e and Supplementary Fig. 48), manifesting that nearly all the N sources were converted into NH_3_ (Supplementary Fig. 49). Comparatively, the NO_3_^‒^ to NH_3_ conversion efficiency of W-MoS_2_ and MoS_2_ were as low as ~ 16% and ~ 30%, and the residual NO_3_^‒^ still remained a relatively high level of 4.64 mM and 11.78 mM even after 48 h (Supplementary Fig. 50). Also, Ag-MoS_2_ behaved with stable performance and robust structure, as demonstrated by the steady eight-cycling NO_3_RR process at 25 °C (Fig. 5f), the negligible morphology change, tiny phase structure difference, and slight chemical valence state shift after the electrolysis (Supplementary Figs. 51, 52).

Furthermore, we demonstrated the practicality of the proposed setup for efficient NH_3_ collection. Here, we integrated electrocatalysis with an air-stripping method for the recovery of high-purity ammonia products at − 1.0 V *versus* RHE (Fig. 5g). After the NO_3_RR process, the cathodic electrolyte was transferred to a conical flask and adjusted to an alkaline state. Due to the high NH_3_ vapor pressure under alkaline conditions, the produced NH_3_ was efficiently extracted at 70 °C using an air stripping method (see details in Methods)^73,74^. Consequently, approximately 98.9% of the NH_3_ vapor was successfully stripped out from the electrolyte (Fig. 5h), indicating favorable NO_3_^‒^ conversion and simultaneous high-efficiency NH_3_ generation. Subsequently, around 97.3% of the outflowing NH_3_ gas was collected in an HCl solution, and approximately 97.1% of NH_4_Cl powder was finally obtained after rotary evaporation. The high-purity NH_4_Cl products were confirmed by XRD measurement (Supplementary Fig. 53), highlighting their potential as fertilizer for agricultural production.

Next, to investigate the practical application prospect, complex electrolyte (which included CO_3_^2‒^, Na^+^, Cl^‒^, K^+^, NO_3_^‒^, and SO_4_^2‒^) was prepared to simulate the nitrate-containing wastewater condition^75^. The NH_3_ Faradaic efficiency over Ag-MoS_2_ in simulated wastewater was 76.7%, while the NH_3_ yield rate reached 16.02 mg h^−1^ cm^−2^ (Supplementary Fig. 54a). The decreased NO_3_RR performance can be ascribed to the presence of interfering ions that influenced the targeted adsorption of NO_3_^‒^. Although the NO_3_RR performance in wastewater decreased to a certain extent, the NH_3_ yield rate and Faradaic maintained 80% and 76% of the optimal performance for simple electrolyte. In the recovery process, approximately 73.9% of NH_4_Cl powder was finally obtained (Supplementary Fig. 54b). Also, we have evaluated the preparation cost of Ag-MoS_2_ catalyst, whose price with 1 cm^2^ was as low as 0.475 ¥ (Supplementary Table 8). These results suggested that the Ag-MoS_2_ has the potential for the conversion of NO_3_^‒^ to ammonia product in actual industrial and agricultural wastewater.

**Fig. 6:**
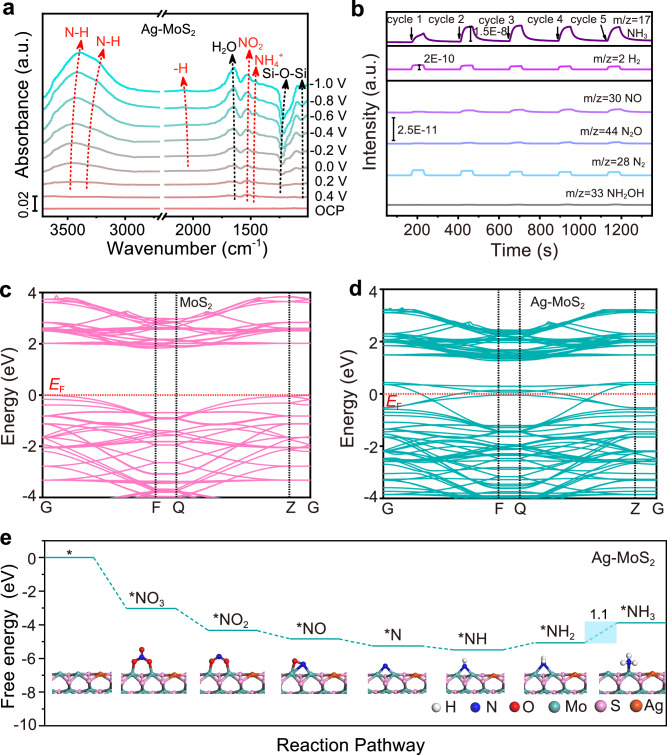
Mechanistic study on NO_3_^–^ electroreduction. **a** In situ ATR-IR spectra of Ag-MoS_2_. The Si-O signal was derived from the reduction of surface SiO_2_ on the Si semi-cylindrical prism substrate under the applied potentials. **b** Online differential electrochemical mass spectrometry (DEMS) measurements of NO_3_RR over Ag-MoS_2_ under the potential of −0.6 V *versus* RHE. Electronic band structures of (**c**) MoS_2_ and (**d**) Ag-MoS_2_. **e** Gibbs free energy diagram of various intermediates generated during NO_3_RR over Ag-MoS_2_ at pH = 7.

To gain a deeper understanding of the NO_3_RR mechanism over Ag-MoS_2_ catalysts, we employed in situ attenuated total reflection infrared spectroscopy (ATR-IR) to capture intermediates and monitor the reaction. As the applied potential ranged from 0.4 to − 1.0 V *versus* RHE (Fig. 6a), the detected NO_2_ peaks (at ~ 1530 cm^‒1^)^76^ in the spectra of Ag-MoS_2_ indicated the deoxygenation of NO_3_^‒^. The pronounced characteristic peaks of N-H (at 3200–3380 cm^‒1^)^77–79^, -H (at ~ 2050 cm^‒1^)^80^, and NH_4_^+^ (at ~ 1450 cm^‒1^)^81^ verified the effective hydrogenation of nitrogen oxide intermediates to ammonia on a highly protonated surface. In comparison, the NO_2_, -H, and NH_4_^+^ signals were not prominent in the spectra of MoS_2_ and W-MoS_2_ (Supplementary Figs. 55, 56), indicating that the relatively slow reaction rates of the two catalysts resulted in less accumulation of related species on their surfaces. Online differential electrochemical mass spectrometry (DEMS) was further performed to detect molecular intermediates and products (Fig. 6b and Supplementary Figs. 57, 58). The mass-to-charge ratio (m/z) signals of NH_3_ (17), H_2_ (2), N_2_ (28), NO (30), NH_2_OH (33), and N_2_O (44) were detected and tracked. Among the three catalysts, the NH_3_ intensity was the strongest, while other signals (H_2_, N_2_, NO, and N_2_O) shrunk over Ag-MoS_2_, which demonstrated the promotion of NH_3_ generation under the S-L junction-induced NO_3_^‒^ enrichment on the Ag-MoS_2_ surface. Further, we calculated the electronic band structures and partial density of states (PDOS) through density functional theory (DFT). After Ag doping, the newly emerged band tail states at VBM extended the valence band of Ag-MoS_2_ (Fig. 6c, d and Supplementary Fig. 59)^82,83^, leading to the downward shift of the Fermi level. While, W doping induced upward shift of the Fermi level away from the VBM (Supplementary Fig. 60). These phenomena were in consistent with UPS and M-S results, unveiling that Ag doping benefited the downward band bending within S-L junction and thus the charge transfer between IHP and the catalyst. The optimal reaction pathways of Ag-MoS_2_ were also calculated (*NO_3_ → *NO_2_ → *NO → *N → *NH → *NH_2_ → *NH_3_) by comparing multiple possible branches (Fig. 6e and Supplementary Figs. 61–63). All three samples underwent the process of adsorption of NO_3_^–^, deoxygenation of the N species, and hydrogenation of the N species to synthesize NH_3_, in which the *NH_2_ → *NH_3_ process was the potential-determining step (PDS). The Gibbs free energy change (ΔG) of this step was 1.1, 0.95, and 1.02 eV for Ag-MoS_2_, MoS_2_, and W-MoS_2_ catalysts at pH = 7. The ΔG of Ag-MoS_2_ was comparable to those of W-MoS_2_ and MoS_2_, suggesting that Ag doping had little contribution to the ΔG of PDS. Although MoS_2_ and W-MoS_2_ exhibited reduced ΔG of PDS at corresponding optimal NO_3_^–^ adsorption potentials (Supplementary Figs. 61 and 64, 65), while, their NO_3_RR performance were far below Ag-MoS_2_ (Fig. 5a). These results further indicated the decisive role of Ag doping-induced surface NO_3_^–^ enrichment effect in promoting NO_3_RR activity in low concentration system.

## Discussion

In summary, we proposed a strategy to enrich charged chemicals in the vicinity o of the electrodes through catalyst bandstructure engineering, overcoming the bottleneck of mass transfer posed by intrinsic charge repulsion in electrocatalysis. We systematically demonstrated the S-L junction-mediated charge rearrangement effect on the IHP region, boosting electrocatalytic reduction of low-concentration NO_3_^–^ to NH_3_. COMSOL simulations and experimental measurements, including in situ Raman spectra, IC, EIS, and ACV, elucidated that the construction of S-L junction induced positively charged IHP, significantly enriching NO_3_^–^ anions concentration (by a factor of 27.6) to break the mass transfer limitation at the reduction potential.

As a proof of concept, an optimized Ag-MoS_2_ was introduced as a model platform for the NO_3_RR flow cell system. We evaluated the comprehensive performance through the decontamination of nitrate polluted solution into drinkable level, which requires efficient catalytic process within a broad range of concentration and high conversion rate. By virtue of the unparalleled performance at ultra-low concentrations (~ 20 mg h^‒1^ cm^‒2^, FE of nearly 100% under concentration of 10 mM), the catalyst with S-L junction succeeded in removing NO_3_^–^ to low level within 3 h, while the counterpart without the junction cannot meet the level after treatment of 48 h. Considering the practicality of the utilization of the high-value-added ammonia product, we implement additional experimental efforts to show a near-unity efficiency by the conversion of NO_3_^–^ to a high-purity NH_4_Cl, aiming to achieve sustainable development.

Efficient anion accumulation on the reaction interface is crucial for a wide range of renewable electricity-driven reduction applications. Thus, the strategy we proposed holds promise for various electrochemical anion reduction reactions, including NO_3_^–^, NO_2_^–^, AsO_4_^3–^, and Cr_2_O_7_^2–^, etc, inspiring innovative design for water treatment and remediation in an environmentally friendly way. Such investigations could illuminate avenues for both fundamental research and practical applications across various scientific domains, ranging from physics, chemistry to environmental sciences.

## Methods

### Chemicals

Sodium molybdate dehydrate (Na_2_MoO_4_ · 2H_2_O, ≥ 99.95%), thiourea (CH_4_N_2_S, ≥ 99%), silver nitrate (AgNO_3_, ≥ 99.8%), hydroxylamine hydrochloride (NH_2_OH·HCl, ≥ 99.99%), hexadecyl trimethyl ammonium bromide (C_19_H_42_BrN, CTAB, ≥ 99%), sodium tungstate dihydrate (Na_2_WO_4_ · 2H_2_O, ≥ 99.5%), salicylic acid (C_7_H_6_O_3_, ≥ 99%), trisodium citrate dihydrate (Na_3_C_6_H_5_O_7_·2H_2_O, ≥ 99%), sulfanilamide (C_6_H_8_N_2_O_2_S, ≥ 99.8%), *p-*dimethylaminobenzaldehyde ((CH_3_)_2_NC_6_H_4_CHO, PDAB, ≥ 98%), sodium nitroferricyanide (III) dihydrate (Na_2_Fe(CN)_5_NO · 2H_2_O, ≥ 99.98%), N-(1-Naphthyl) ethylenediamine dihydrochloride (C_12_H_14_N_2_·2HCl, ≥ 98%), potassium sulfate (K_2_SO_4_, ≥ 99%), potassium nitrate-^15^N (^15^KNO_3_, ≥ 99%), dimethyl sulfoxide (C_2_H_6_SO, DMSO-*d*6, ≥ 99.9%), maleic acid (C_4_H_4_O_4_, ≥ 99%), and ammonium chloride (^14^NH_4_Cl, ≥ 99.8%), ammonium-^15^N chloride (^15^NH_4_Cl, ≥ 98.5%) were purchased from Aldrich Chemical Reagent Co., Ltd. Sodium hydroxide (NaOH, ≥ 96%), sodium hypochlorite (NaClO, analytical pure), hydrochloric acid (HCl, 38%), potassium nitrate (KNO_3_, ≥ 99.999%), potassium nitrite (KNO_2_, ≥ 97%), and ethanol (C_2_H_6_O, ≥ 99.7%) were purchased from Sinopharm Chemical Reagent Co., Ltd. All chemicals were used as received without further purification. The water used in this research was purified through a Millipore system.

### Preparation of Ag-MoS_2_

For the synthesis of Ag-MoS_2_ in a typical hydrothermal process, 4.27 mmol of Na_2_MoO_4_ · 2H_2_O, 18.40 mmol of CH_4_N_2_S, a certain amount of AgNO_3_, and 10.43 mmol of NH_2_OH · HCl were dissolved in 50 mL of deionized water under vigorous stirring for 0.5 h. 0.18 g of CTAB was then added to the mixed solution, and the pH value of the mixture was adjusted to 6. After that, carbon cloth with an area of 4 × 4 cm^2^ and a thickness of 0.35 mm was immersed into the homogeneous solution. The mixture was subsequently transferred into a 100 mL Teflon-lined stainless-steel autoclave and heated at 180 °C for 24 h in an oven. After cooling down to room temperature naturally, the products were washed with ethanol and deionized (DI) water repeatedly, followed by vacuum-freeze drying. The obtained sample was Ag-MoS_2_ grown on carbon cloth (catalyst mass of ~ 5 mg cm^−2^). Ag-MoS_2_ catalysts with various Ag-doped amounts were prepared according to the above procedure by changing the additional amount of AgNO_3_. Specifically, 0.213 mmol, 0.426 mmol, 0.639 mmol and 0.852 mmol AgNO_3_ were added to achieve 1.87 wt%, 3.49 wt%, 5.91 wt% and 6.91 wt% Ag doping amounts, respectively. These catalysts were denoated as Ag-MoS_2_-5, Ag-MoS_2_-10, Ag-MoS_2_-15 and Ag-MoS_2_-20 (Supplementary Table S1).

### Preparation of MoS_2_

In a typical synthesis of MoS_2_, 4.27 mmol of Na_2_MoO_4_ · 2H_2_O, 18.40 mmol of CH_4_N_2_S, and 10.43 mmol of NH_2_OH · HCl were dissolved in 50 mL of deionized water under vigorous stirring for 0.5 h. 0.18 g CTAB was then added to the mixed solution, and the pH value of the mixture was adjusted to 6. After that, carbon cloth with an area of 4 × 4 cm^2^ and a thickness of 0.35 mm was immersed into the homogeneous solution. The mixture was subsequently transferred into a 100 mL Teflon-lined stainless-steel autoclave and heated at 180 °C for 24 h in an oven. After cooling down to room temperature naturally, the products were washed with ethanol and DI water repeatedly, followed by vacuum-freeze drying. The obtained sample was MoS_2_ grown on carbon cloth (catalyst mass of ~ 5 mg cm^−2^).

### Preparation of W-MoS_2_

To systhesize W-MoS_2_, 4.27 mmol of Na_2_MoO_4_ · 2H_2_O, 18.40 mmol of CH_4_N_2_S, 0.426 mmol of Na_2_WO_4_ · 2H_2_O, and 10.43 mmol of NH_2_OH · HCl were dissolved in 50 mL of deionized water under vigorous stirring for 0.5 h. 0.18 g of CTAB was then added to the mixed solution, and the pH value of the mixture was adjusted to 6. After that, carbon cloth with an area of 4 × 4 cm^2^ and a thickness of 0.35 mm was immersed into the homogeneous solution. The mixture was subsequently transferred into a 100 mL Teflon-lined stainless-steel autoclave and heated at 180 °C for 24 h in an oven. After cooling down to room temperature naturally, the products were washed with ethanol and DI water repeatedly, followed by vacuum-freeze drying. The obtained sample was W-MoS_2_ grown on carbon cloth (catalyst mass of ~ 5 mg cm^−2^).

### Electrochemical testing

Before the NO_3_RR tests, the pretreatment of Nafion 117 membrane (area: a circular shape with a diameter of 2 cm and a thickness of ~ 183 μm) was as follows: the membrane was first oxidized in 5% H_2_O_2_ solution at 80 °C for 1 h, next boiled in DI water for 1 h, then used 0.5 M H_2_SO_4_ at 80 °C for 1 h, finally washed with DI water.

The NO_3_RR tests were performed on an electrochemical workstation (PARSTAT 4000) with a typical three-electrode flow cell, including as-prepared catalyst electrode (area of 1 × 1 cm^2^, catalyst mass of ~ 5 mg cm^−2^), IrO_2_ electrode (area of 1 × 1 cm^2^), and a saturated calomel electrode as the working electrode, counter electrode, and reference electrode, respectively. Nafion 117 membrane was used to separate the anodic cell and cathodic cell. The flow rate of electrolyte (0.5 M K_2_SO_4_ with 10 mM NO_3_^‒^) was set at 2 mL min^‒1^ in cathodic and anodic chambers. All potentials reported in this work were referred to the RHE scale via calibration by the following equation: ${\rm{E}}({\rm{versus}}\,{\rm{RHE}})={\rm{E}}({\rm{versus}}\,{\rm{SCE}})+0.244+0.0591\times {\rm{pHvalue}}$. The three batches of catalysts (Ag-MoS_2_ (AgNO_3_ amount of 0.426 mmol), MoS_2_ and W-MoS_2_) were synthesized, respectively, and their NO_3_RR performance at various potentials were tested. The error bars were the mean values standard deviation according to the obtained data. For the chronoamperometry measurement, the potential was applied from − 0.2 to − 1.0 *versus* RHE. LSV was carried out in a voltage window from 0.25 to − 1.65 V *versus* RHE at scan rates of 10 mV·s^−1^. EIS of the samples was performed at the frequency region from 10^6^ to 10^−2 ^Hz with an amplitude of 20 mV at various applied potentials. During the electrochemical testing process, no iR compensation was performed. Mott-Schottky measurements were carried out at the frequency of 1000 Hz under dark conditions to obtain the flat band potential and carrier concentration of the samples, in which the dielectric constant of MoS_2_ was 7.6^75,84^.

### Detection and quantification of NH_3_ using UV-vis

The concentration of NH_3_ after chronoamperometry measurements with different potentials was detected by the salicylic acid method^85^. The cathodic electrolyte after electrolysis was collected and diluted to the detection range. Then, 2 mL of diluted liquid was added to 2 mL of 1 M NaOH solution containing 5 wt% C_7_H_6_O_3_ and 5 wt% Na_3_C_6_H_5_O_7_·2H_2_O. 1 mL of 0.05 M NaClO and 0.2 mL of C_5_FeN_6_Na_2_O solution (1 wt%) were subsequently mixed with the aforementioned solution and shaken well. After standing for 2 h, UV-vis spectrophotometer measurements were performed with the range from 500 to 800 nm, and the absorbance value at the wavelength of 655 nm was recorded. The concentration-absorbance curve was calibrated using standard ammonium chloride solution with concentrations of 0.5, 1.0, 3.0, 5.0, 10.0, 20.0 and 30.0 µg mL^−1^ in 0.5 M K_2_SO_4_ with NO_3_^‒^. The fitting curve $(y=0.10x+0.01,{{\rm{R}}}^{2}=0.999)$ exhibited a good linear relation of absorbance value with NH_4_^+^ concentration.

### Detection and quantification of NO_3_^‒^ using UV-vis

The cathodic electrolyte after electrolysis was collected and diluted to the detection range. In the process, 5 mL of diluted sample solution was mixed with 0.1 mL of 1 M HCl. After standing for 20 min, the UV-vis absorbance at the wavelength ranging from 215 to 280 nm was recorded^86,87^. The absorbance difference between the wavelengths at 220 and 275 nm was calculated using the equation: ${\rm{A}}={{\rm{A}}}_{220\,{\rm{nm}}}-{{\rm{A}}}_{275\,{\rm{nm}}}$. The concentration-absorbance difference curve was calibrated using standard KNO_3_ solution with 1, 5, 10, 20, 30 and 50 µg mL^−1^ concentrations. The fitting curve $(y=0.034x-0.036,{{\rm{R}}}^{2}=0.999)$ exhibited a good linear relation of absorbance value with NO_3_^‒^ concentration.

### Detection and quantification of NO_2_^‒^ using UV-vis

The preparation of the color reagent was as follows^86,87^: First, 0.5 g of C_6_H_8_N_2_O_2_S was dissolved in 50 mL of 2.0 M HCl solution, which was labeled as reagent A. 20 mg of N-(1-naphthyl) ethylenediamine dihydrochloride was then dispersed in 20 mL of DI water, which was labeled as reagent B. After 0.1 mL of reagent A mixed with 5 mL of standard or diluted sample solutions, 0.1 mL of reagent B was finally injected into the homogeneous solution, shaking up and standing for 30 min. The UV-vis absorbance at the wavelength ranging from 400 to 640 nm was recorded, in which the characteristic absorption peak of NO_2_^‒^ was identified at 540 nm. The concentration-absorbance difference curve was calibrated using standard KNO_2_ solution with concentrations of 0.2, 0.5, 1.0, and 2.0 µg mL^−1^. The fitting curve $(y=0.446x+0.039,{{\rm{R}}}^{2}=0.999)$ exhibited a good linear relation of absorbance value with NO_2_^‒^ concentration.

### Detection and quantification of NH_3_ using ^1^H NMR

^14^NO_3_^‒^ and ^15^NO_3_^‒^ isotope labeling experiments were carried out on a Bruker AVANCE III HD NMR spectrometer (600 MHz) to support the UV-vis results^12^. After electrolysis, the pH value of the cathodic electrolyte was adjusted to 2 with 1 M HCl. 0.5 mL of the homogeneous solution was then mixed with 0.1 mL DMSO-*d*6 with 0.04% C_4_H_4_O_4_. ^1^H NMR was recorded to quantitatively determine NH_3_ concentration according to the corresponding standard curves.

#### Electrochemical in situ Raman spectroscopy

In situ Raman spectroscopy was measured by inVia Reflex (Renishaw, UK) with a 532 nm laser as the excitation source^20^. The electrochemical process was performed in the three-electrode custom-made Teflon reactor with a quartz window, including Ag/AgCl, Pt wire, and catalysts coated on the Au electrode as the reference electrode, counter electrode, and working electrode, respectively. The spectra were recorded by the potential from OCP to − 0.6 V *versus* RHE.

#### Electrochemical in situ ATR-IR spectroscopy

ATR-IR spectroscopy was tested on a Nicolet iS50 FT-IR spectrometer equipped with an MCT detector and cooled by liquid nitrogen^20^. The electrochemical process was performed in the three-electrode custom-made reactor, including Ag/AgCl and Pt wire as the reference electrode and counter electrode, respectively. The working electrode was prepared as follows: The Si semi-cylindrical prism was first polished with Al_2_O_3_ powder and sonicated in acetone and deionized water. The Si was placed in a piranha solution at 60 °C for 20 min to remove the organic contaminants. Then the reflecting surface of Si was deposited in the Au precursor mixture at 60 °C for 10 min, and the Au-coated Si conductive substrate was obtained. The catalyst ink was finally coated on the substrate-reflecting surface. After that, the spectra were recorded by the potential from 0.4 V to − 1.0 V *versus* RHE. The spectrum collected at OCP was as background subtraction.

#### Electrochemical online DEMS tests

The online DEMS tests^21^ were measured in the three-electrode customized reactors containing 0.5 M K_2_SO_4_ with 10 mM NO_3_^‒^ electrolyte, including catalysts coated on breathable film with Au plating layer, Pt wire, and saturated Ag/AgCl electrode as the working electrode, the counter electrode and the reference electrode, respectively. The potential of OCP and − 0.6 V *versus* RHE were applied alternately with an interval of 3 min after the baseline of the mass spectrometry kept steady. During the electrochemical process, the differential mass signals were recorded when the gaseous products formed on the electrode surface. The operation process was repeated five cycles to avoid accidental errors.

#### Direct ammonia product synthesis

By coupling NO_3_RR with the Ar stripping process, the cathodic electrolyte after electrolysis was adjusted to alkaline state, and sealed in a conical flask at 70 °C with flowing 100 sccm Ar gas to purge the NH_3_ gas out. The outlet stream was injected into 2 M HCl to capture the NH_3_ product. The NH_3_ amount in all the solutions was evaluated by the salicylic acid method mentioned above, and the removal efficiency and collection efficiency were calculated as following equations^12^, respectively:

1 $${{\rm{Removed}}\,{\rm{NH}}}_{3}\,{\rm{via}}\,{\rm{Ar}}\,{\rm{stripping}}=1-\frac{{{\rm{NH}}}_{3}\,{\rm{left}}\,{\rm{after}}\,{\rm{Ar}}\,{\rm{stripping}}\,({\rm{mol}})}{{{\rm{initial}}\,{\rm{NH}}}_{3}\,({\rm{mol}})}$$

2 $${{\rm{Acid}}\,{\rm{collected}}\,{\rm{NH}}}_{3}=\frac{{{\rm{NH}}}_{3}\,{\rm{in}}\,{\rm{acid}}\,{\rm{trap}}\,({\rm{mol}})}{{{\rm{removed}}\,{\rm{NH}}}_{3}\,{\rm{via}}\,{\rm{Ar}}\,{\rm{stripping}}\,({\rm{mol}})}$$

To estimate the efficiency of the NH_4_Cl product, the HCl solution with the trapped NH_3_ was dried by rotary evaporator at 70 °C. The generated NH_4_Cl was measured by a balance and analyzed by XRD. The collection efficiency of NH_4_Cl from the acid trap was calculated by the following equation:

3 $${{\rm{Collected}}\,{\rm{NH}}}_{4}{\rm{Cl}}\,{\rm{from}}\,{\rm{acid}}\,{\rm{trap}}=\frac{{{\rm{collected}}\,{\rm{dried}}\,{\rm{out}}\,{\rm{NH}}}_{4}{\rm{Cl}}\,({\rm{mol}})}{{{\rm{acid}}\,{\rm{collected}}\,{\rm{NH}}}_{3}\,({\rm{mol}})}$$

#### DFT calculations

All calculations were carried out by spin-polarized DFT with the Vienna Ab initio Simulation Package (VASP)^88,89^. Electron exchange-correlation was expressed by the Perdew-Burke-Ernzerhof (PBE) functional within the generalized gradient approximation (GGA)^90^. To describe the ionic cores, the projector augmented wave (PAW) pseudopotential was applied^91,92^. The plane wave energy cutoff, and convergence criterion for electronic energy and forces were set as 450 eV, 10^−5^ eV, and 0.02 eV/Å. van der Waals (VDW) interactions were corrected using the D3 method of Grimme^93^. To compare the electronic structures of MoS_2_, Ag-MoS_2_ and W-MoS_2_, we constructed a 2 × 2 × 2 MoS_2_ supercell containing 16 Mo atoms and 32 S atoms. Substituting one Mo atom with an Ag atom is referred to as Ag-MoS_2_, while substituting one MoS_2_ atom with a W atom is referred to as W-MoS_2_. For investigating the reaction pathways of nitrate ions on three catalytic surfaces, we built a nanoribbon containing 20 Mo atoms and 40 S atoms. The bottom two layers were fixed to simulate the edge of a single-layer MoS_2_. For Ag-MoS_2_ and W-MoS_2_, we replaced one edge Mo atom with an Ag atom and a W atom, respectively. To avoid periodic interlayer interactions, we introduced a vacuum layer of 20 Å in the Z and Y directions. Aqueous phase H_2_O and NO_3_^‒^ were as the energetics references.

## Supplementary information

Supplementary Information

Description of Additional Supplementary Files

Supplementary Data 1

Peer Review File

## Source data

Source Data

## Data Availability

Full data supporting the findings of this study are available within the article and its Supplementary Information, as well as from the corresponding author upon reasonable request. Source data are provided in this paper.
