# Supplementary material for: Near-Unity Nitrate to Ammonia conversion via reactant enrichment at the solid-liquid interface
Source: Nat Commun. 2025 Jul 1;16:5715. doi: 10.1038/s41467-025-60671-y (PMC12219233; doi:10.1038/s41467-025-60671-y)
Supplement: Supplementary file 1 — Supplementary Information [file 41467_2025_60671_MOESM1_ESM.pdf]

Supplementary information

Near-Unity Nitrate to Ammonia Conversion via Reactant Enrichment at the Solid-Liquid Interface

Wanru Liao[1,2,+], Jun Wang[1,2,+], Yao Tan[1], Xin Zi[1], Changxu Liu[3], Qiyou Wang[1], Li Zhu[4], Cheng-Wei Kao[5], Ting-Shan Chan[5], Hongmei Li[1], Yali Zhang[6], Kang Liu[1], Chao Cai[1], Junwei Fu[1], Beidou Xi[7,*], Emiliano Cortés[4,*], Liyuan Chai[8], Min Liu[1,8,*]

[1] Hunan Joint International Research Center for Carbon Dioxide Resource Utilization, State Key Laboratory of Powder Metallurgy, School of Physics, Central South University, Changsha 410083, Hunan, P. R. China.

[2] School of Chemistry and Pharmaceutical Engineering, Changsha University of Science and Technology, Changsha 410114, Hunan, P. R. China.

[3] Centre for Metamaterial Research & Innovation, Department of Engineering, University of Exeter, Exeter EX4 4QF, UK.

[4] Nanoinstitut München, Fakultät für Physik, Ludwig-Maximilians-Universität München, München 80539, Germany.

[5] National Synchrotron Radiation Research Center, Hsinchu 300092, Taiwan.

[6] Key Laboratory of Land Surface Pattern and Simulation, Institute of Geographic Sciences and Natural Resources Research, Chinese Academy of Sciences, 100101, Beijing, P. R. China.

[7] State Key Laboratory of Environmental Criteria and Risk Assessment, Chinese Research Academy of Environmental Sciences, 100012, Beijing, P. R. China.

[8] School of Metallurgy and Environment, Central South University, Changsha 410083, P. R. China.

[+] These authors contributed equally to this work.

Corresponding authors: xibd@craes.org.cn; Emiliano.Cortes@lmu.de; minliu@csu.edu.cn

Experimental Section:

Calculation of the Faradaic efficiency and the yield rate for NH_3.

The NH_3 Faradaic efficiency for NO_3RR could be calculated as follows:

$$FE(NH_3) = (n \times F \times c_{NH3} \times V) / (M \times Q) \quad (1)$$

The NH_3 yield rate for NO_3RR could be calculated as follows:

$$\text{Yield rate}(NH_3) = (c_{NH3} \times V) / (t \times A) \quad (2)$$

Where n is the electron-transfer number (for 1 mol NH_3, it was 8), F was the Faradaic constant (96,485 C mol^{-1}), c_{NH3} was the measured NH_3 concentration (μg mL^{-1}), V was the volume of the cathode electrolyte (60 mL), M was the relative molecular mass of products (17), Q was the applied overall coulomb quantity (C), A was the area of the catalyst electrode (1 cm^2), and t was the reaction time (0.5 h).

ECSA analysis.

The electrochemical double-layer capacitance (C_{dl}) of the materials was tested to determine their electrochemical surface area (ECSA) using the cyclic voltammetry (CV) in non-faradic regions with diverse scan rates ranging from 20 to 100 mV s^{-1} between 0.62 and 0.72 V *versus* RHE. The plotted current density (difference between the anode current density and cathode current density at 0.66 V *versus* RHE) against scan rate has shown a linear relationship and its slope was twice the C_{dl}. The ECSA was determined by:

$$ECSA = C_{dl} / C_s \quad (3)$$

The surface-area-normalized activity of NH_3 could be calculated as below:

$$\text{Yield rate}_{ECSA}(NH_3) = (c_{NH3} \times V) / (t \times ECSA) \quad (4)$$

where C_s was the specific capacitance of the sample, a general specific capacitance of $C_s = 0.040$ mF cm^{-2} was used in this study. c_{NH3} was the measured NH_3 concentration (μg mL^{-1}), V was the volume of the cathode electrolyte (60 mL), and t was the reaction time (0.5 h).

Calculation of nitrate conversion and product selectivity.

The conversion of NO_3^- could be calculated as below:

$$C(NO_3^-)\% = (C_0(NO_3^--N) - C_t(NO_3^--N)) / C_0(NO_3^--N) \times 100\% \quad (5)$$

The conversion of NO_3^- could be calculated as below:

$$S(NO_2^-)\% = C_t(NO_2^--N) / (C_0(NO_3^--N) - C_t(NO_3^--N)) \times 100\% \quad (6)$$

$$S(NH_4^+)\% = C_t(NH_4^+-N) / (C_0(NO_3^--N) - C_t(NO_3^--N)) \times 100\% \quad (7)$$

Where $C_0(NO_3^--N$ (mg L^{-1}) and $C_t(NO_3^--N)$ (mg L^{-1}) were the initial nitrate concentration and the nitrate concentration at different electrolysis time, respectively. $C_t(NO_2^--N)$ and $C_t(NH_4^+-N)$ were the concentrations of nitrite and ammoniums at different electrolysis time.

Quantitative analysis of built-in electric field

The calculation of the electric field intensity on the electrode side within S-L junction is based on the following equation.[1-3]

$$F_s = (-2V_s\rho/\varepsilon\varepsilon_0)^{1/2} \quad (8)$$

Where F_s is the internal electric field magnitude, V_s is the surface voltage, ρ is the surface charge density, ε is the low-frequency dielectric constant, and ε_0 is the permittivity of free space. As ε and ε_0 are constants, the internal electric field intensity is primarily determined by the surface voltage and the surface charge density.

The surface voltages of the samples were obtained by the open-circuit potential measurement of the samples under chopped light condition. As Le Formal and Gratzel et al. reported, the accumulated charge on the surface is proportional to the integral value, which is calculated from the transient photocurrent density minus the steady-state photocurrent density at the same time. Therefore, the transient photocurrent density measurements were conducted. The surface charge densities of the as-prepared samples under operating condition were then obtained by the integral of the transient anodic photocurrent peaks at negative bias condition (-0.6 V *versus* RHE).

Adsorbed NO_3^- measurement by ion chromatography

NO_3^- adsorption capacity of the catalysts was characterized by Ion Chromatography (IC, Thermo Scientific ICS-600 Ion Chromatography System). The catalyst electrode was performed in a 10 mM NO_3^- electrolyte with a constant potential at -0.6 V *versus* RHE for 120 s, and then removed into 10 mL ultrapure water and shook for 20 s, repeating the above steps 3 times to capture the NO_3^- adsorbed on the surface of the electrode. Finally, the concentration of NO_3^- in ultrapure water was quantitatively determined by ion chromatography with standard curves.

COMSOL multiphysics simulations

The finite element method (FEM) simulations were conducted through COMSOL Multiphysics v 6.0. The Electric Currents (ec) and Transport of Diluted Species (tds) physics modules in COMSOL Multiphysics were used to simulate and calculate the performance of the models in electrolytes. All the meshes in the model were set to free tetrahedral meshing. The relative tolerance in the steady-state solver was set to 0.01. The presented simulation results are based on the steady-state solver.

The Electric Current was used to simulate the distribution of electric field with applied voltage. The formula for calculating the electric field as follows:

$$E = -\nabla V \quad (9)$$

And the dielectric model follows the rules:

$$D = \varepsilon_0 \varepsilon_r E \quad (10)$$

Where ε_0 is the dielectric constant of vacuum, ε_r is the dielectric constant of the materials.

The distribution of NO_3^- is dynamically balanced by the combined action of electric field and ion diffusion, following the material transfer equation and the Ernest equation:

$$\nabla \cdot J_j = R_j \quad (11)$$

$$J_j = -D_j \nabla c_j - z_j u_{m,j} F c_j \nabla V \quad (12)$$

Where J_j is the total flux of the substance, R_j is the reactive source of the

electrolyte solution, D_j is the diffusion coefficient, c_j is the concentration of both ions, z_j is the number of charges, $u_{m,j}$ is the migration rate, F is the faradaic constant, V is the potential of the electrode in the electrolyte.

According to the trend of experimental values, different potential is applied to the electrode surface to simulate the concentration of nitrate ion on the electrode surface under different conditions. It mainly affects the electromigration of nitrate ions.

The thickness of the diffusion layer was assumed to be 200 μm. For the ion concentration boundary condition, the ion concentration at the upper boundary of the model was set to the concentration of the native solution, and the lower boundary was set to no flux. For the electrostatic field boundary conditions, the upper boundary was set to 0 V, and the lower boundary was set to -0.6, -0.8, and -1.0 V, respectively, depending on the degree of electric field reversal potential built into different materials.

DFT computational details

Calculation of thermodynamic corrections

The zero-point energy for each species is calculate by

$$E_{ZPE}=\sum_i \frac{hv_i}{2} \tag{13}$$

where v_i is the vibration frequency. The entropy contributions of translational, rotational, vibrational, and electronic motion can be calculated by

$$S_t=R\{\ln\left[\left(\frac{2\pi m k_B T}{h^2}\right)^{\frac{3}{2}}\frac{k_B T}{P}\right]+\frac{5}{2}\} \tag{14}$$

$$S_r=R[\ln\left(\frac{T}{\sigma_r}*\frac{8\pi^2 I k_B}{h^2}\right)+1] \tag{15}$$

$$S_v=R\sum_i\{\frac{hv_i}{k_B T}\frac{e^{-\frac{hv_i}{k_B T}}}{1-e^{-\frac{hv_i}{k_B T}}}-\ln[1-e^{-\frac{hv_i}{k_B T}}]\} \tag{16}$$

$$S_e=R*\ln(N+1) \tag{17}$$

where N is the number of unpaired electrons, R is the gas constant, P is the pressure, k_B is the Boltzmann constant[4].

From the above formula, considering that the vibration frequency of the catalyst substrate is small, its corresponding correction is very small and does not affect the

calculation results. Thus, we mainly made corrections to gas molecules and adsorbents on the catalyst, and entropic effects of the catalyst substrate would not be considered further.

H_2O as the proton source in neutral conditions. The pH has an influence on the major proton donor in the research system. For nitrate reaction pathways (pH = 7), we have considered H_2O as the proton source. Under this condition, the H* path will be through the neutral pathway.

$$H_2O + e^- + * \rightarrow H_2O^* + e^- \rightarrow H^* + OH^- \rightarrow 1/2H_2 + OH^- + * \qquad (18)$$

$$G_{OH^-} - G_{H_2O} - G_{e^-} = G_{1/2H_2} - eU_{RHE} \qquad (19)$$

For electrochemical steps in nitrate reduction, the free energy changes are calculated using the products and reactants of the following reaction equation:

$$H_2O + X^* + e^- \rightarrow XH^* + OH^- \qquad (20)$$

$$\Delta G(U) = G_{XH^*} - G_{X^*} + G_{OH^-} - G_{H_2O} - G_{e^-} = G_{XH^*} - G_{X^*} + G_{1/2H_2} - eU_{RHE} \qquad (21)$$

The calculation method for pH and potential effects. The free energies of adsorption of ionic species are calculated using thermodynamic Hess cycles, which cycles include the effects of entropy, solvation energy, protonation energy (including pH effects) and potential effects. The Gibbs energy formulas for nitrate reduction reaction steps are reported by Muhich et. al[5-7]. The method is based on that of Calle-Vallejo et. al[8] and Liu et. al[9]. The free energy of anion A^- is calculated according to:

$$A^- + * \leftrightarrow A^* + e^- \qquad (22)$$

$$\Delta G_{ads}(A^-) = E_{A^*} + [G_{H^+} + G_{e^-}] - [G_{HA} - \Delta G_{sol} - \Delta G_{protonation}] - E_* \qquad (23)$$

Where E_* and E_{A^*} are the DFT computed enthalpies of bare surface and A^* adsorbed to the surface, respectively. G_{H_2} and G_{HA} are the Gibbs free energies of desorbed species H_2 and HA, respectively, in the gas phase at 300 K, as calculated from the following:

$$G_{HA} = E_{HA} + E_{ZPE} - T{*}S \qquad (24)$$

where E_{HA} is the DFT computed energy of HA in the gas phase, T is the temperature (300 K), E_{ZPE} is the contribution of the zero-point energy, S is the entropic

contributions to the free energy obtained using the JANAF database. The solvation energy is described:

$$\Delta G_{sol} = G_{HA(g)} - G_{HA(l)} \quad (25)$$

The pH accounts for the effects on the free energies of the species. Free energy modifications due to pH were calculated according to:

$$\Delta G_{protonation} = G^{o} - 2.303kT(pK_a - pH) = G_{A^-} + G_{H^+} - G_{HA(l)} - 2.303kT(pK_a - pH) \quad (26)$$

$G_{HA(g)}$ and $G_{HA(l)}$ are the free energies of HA molecule in the gas and liquid phases respectively. k is the Boltzmann constant. K_a is the acid dissociation constant for the A^- anion. The standard state (25° C, 100 k*P*a, 1 mol/kg) energies of ion and neutral species in aqueous solution ($G_{HA(g)}$, $G_{HA(l)}$, G_{A^-}, G_{H^+}, K_a) are taken from the CRC handbook. The computational hydrogen electrode (CHE) is used to account for potential effects on reaction energies[10]:

$$\Delta G = \Delta E + \Delta E_{ZPE} - T*\Delta S + 0.0591*pH - eU_{RHE} \quad (27)$$

where ΔE is the DFT computed reaction (electronic) energy, ΔE_{ZPE} and ΔS are the zero-point energy difference and the entropy difference between the adsorbed state and the gas phase, respectively. 0.0591*pH represents the free-energy contribution due to the variations in H concentration. We considered the effect of a potential bias on all states involving one electron or hole in the electrode by shifting the energy of this energy by eU_{RHE}, where U_{RHE} is the electrode potential relative to the reversible hydrogen electrode (RHE).

Characterizations.

X-ray diffraction (XRD) data were recorded over an X'Pert3 Powder diffractometer using Cu Kα radiation (45 kV, 40 mA). The morphology images were studied by scanning electron microscopes (SEM, F EI Helios Nanolab 600i). transmission electron microscopy (TEM) images were obtained from FEI Tecnai G 2 F20 field emission transmission electron microscope operated at 200 kV, equipping with energy dispersive X-ray spectroscopy (EDS) mapping. The chemical state and composition of the samples were characterized using X-ray photoelectron spectroscopy (XPS, Thermo Scientific

K-Alpha) with an Al Kα ($h\nu$ =1486.6 eV) monochrome. All binding energies were referenced to the C 1s peak (284.6 eV) of the surface adventitious carbon. The X-ray absorption spectroscopy (XAS) spectra were measured on a hard X-ray spectrometer at the TLS 01C1 and TLS 16A1 beamlines of the National Synchrotron Radiation Research Center (NSRRC, Taiwan) in the fluorescence mode. The corresponding XAFS data were analyzed through the standard procedures using the Ifeffit package. The Kelvin probe force microscopy (KPFM) experiment was conducted under room temperature and atmospheric pressure. A Bruker icon instrument with an SCM-PIT test probe was utilized. The samples on FTO were scanned at a rate of 0.5 Hz with an image size of 256 × 256. The testing was performed on a vibration isolation table to ensure the accuracy of the results. Ammonia, nitrate, and nitrite were detected by ultraviolet-visible diffuse reflectance spectroscopy (UV-vis) using a UV-2600 spectrophotometer (Shimadzu). Ultraviolet photoelectron spectroscopy (UPS, ThermoFisher ESCALAB 250Xi) was utilized to evaluate the work function of the catalyst. Mott-Schottky measurement was carried out at the frequency of 1000 Hz under the potential of -0.6 V *versus* RHE, to obtain the Fermi level potential of the sample under working condition.

Supplementary Discussions

Fig. 1

Notes: For typical p-type catalyst, the Fermi level position of Ag-MoS_2 is close to the valance band maximum (VBM), which exhibits more positive potential than the redox potential of NO_3^-/NH_3 pair. As the p-type Ag-MoS_2 exhibits the characteristic of hole conduction, when the Ag-MoS_2 is immersed in the NO_3^--containing electrolyte, the potential difference between E_F and E_{redox} induces holes (as the majority carriers in p-type semiconductor) transfer from catalyst surface to the IHP of solution side. As a result, downward band bending occurs on the semiconductor surface, forming space charge layer with hole depletion. And the positively charged IHP could attract nitrate anions under electrostatic interaction.

Supplementary Figures

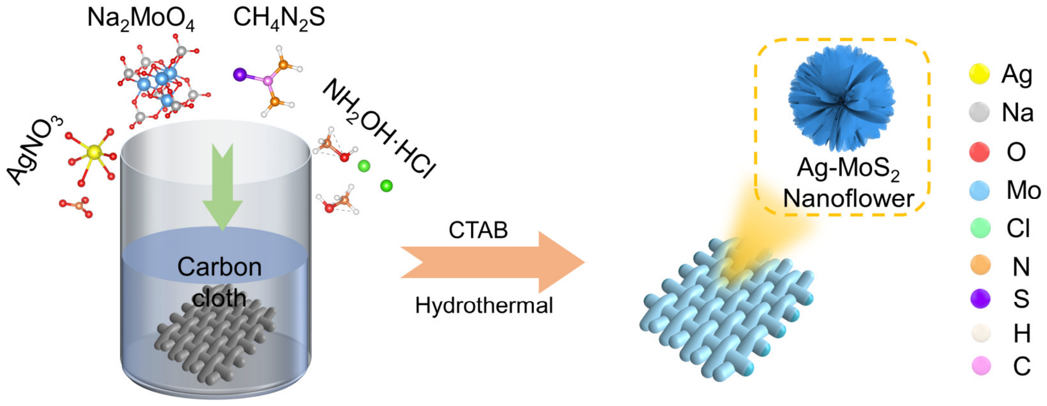

Supplementary Fig. 1 | Catalyst preparation process diagram. The synthetic process of Ag-MoS_2.

Note: Series MoS_2 catalysts were prepared through a facile hydrothermal process. Specifically, Na_2MoO_4 and CH_4N_2S reacted with each other to form Na_2MoS_2 in the first stage. Then, Na_2MoS_2 could be readily reduced to MoS_2 nanoparticles with the help of $NH_2OH·HCl$. Subsequently, the nanoparticles started to assemble together and spontaneously transform to MoS_2 nanosheets. Finally, MoS_2 nanoflowers were formed from several MoS_2 nanosheets with the help of CTAB surfactant. In the present work, carbon cloth with an area of 4 × 4 cm^2 was immersed into the precursor with the addition of $AgNO_3$ as Ag source for the preparation of Ag-MoS_2 catalyst. According to the SEM images (Fig. 2a and Supplementary Fig. 2), the Ag-MoS_2 nanoflowers dispersed evenly on the carbon cloth substrate. Since the hydrothermal reaction is facile and controllable, we consider that the preparation of Ag-MoS_2 with larger scale is feasible.

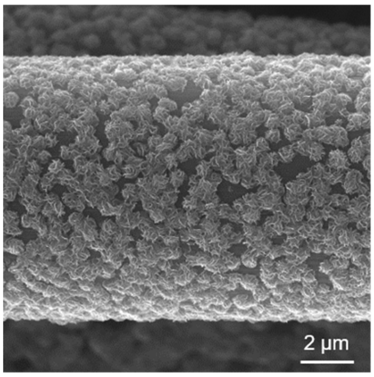

Supplementary Fig. 2 | Morphology characterization of catalyst. SEM image of Ag-MoS_2 grown on carbon cloth.

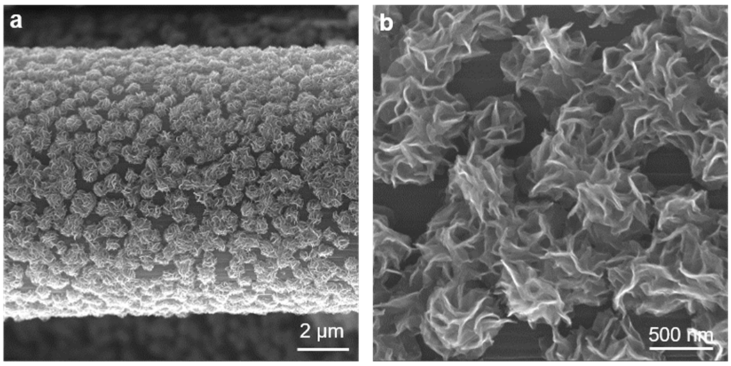

Supplementary Fig. 3 | Morphology characterization of catalyst. SEM images of MoS_2 grown on carbon cloth with the scale bars of (a) 2 μm and (b) 500 nm.

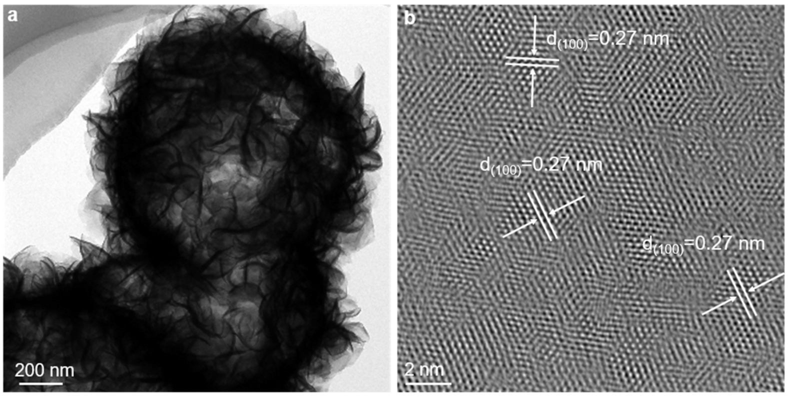

Supplementary Fig. 4 | Structural characterization of catalyst. (a) TEM and (b) HRTEM images of MoS_2.

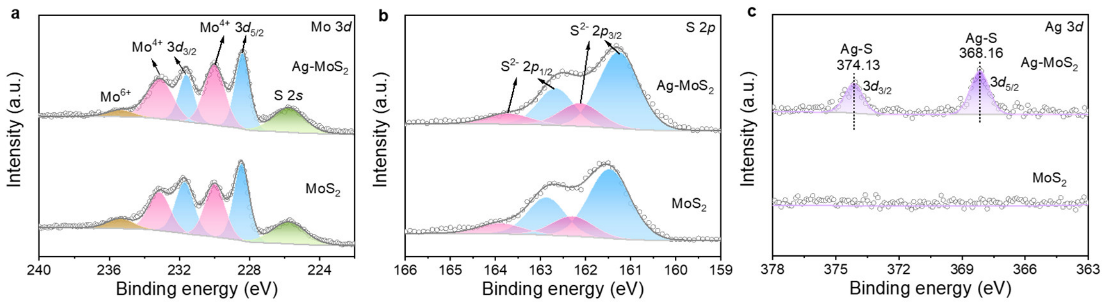

Supplementary Fig. 5 | Valence state analysis of catalyst. (a) Mo 3*d*, (b) S 2*p*, and (c) Ag 3*d* XPS spectra of catalysts.

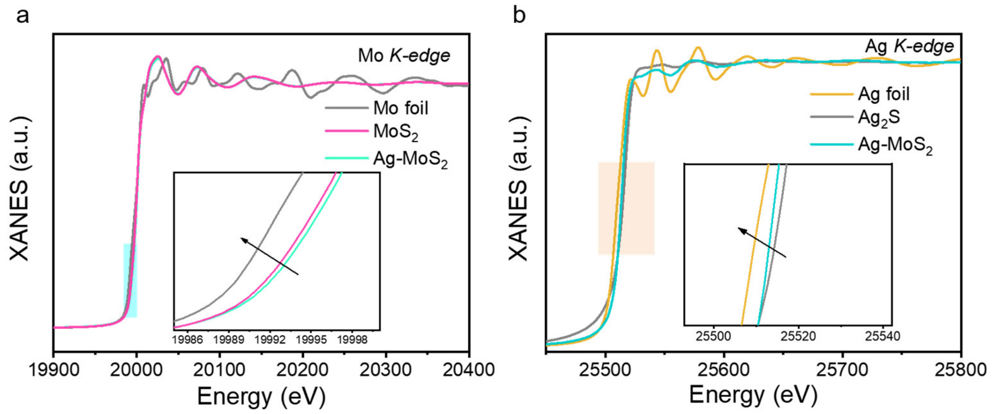

Supplementary Fig. 6 | Electron transfer analysis of catalyst. (a) Mo *K*-edge XANES spectra and (b) Ag *K*-edge XANES spectra of Ag-MoS_2 and reference samples.

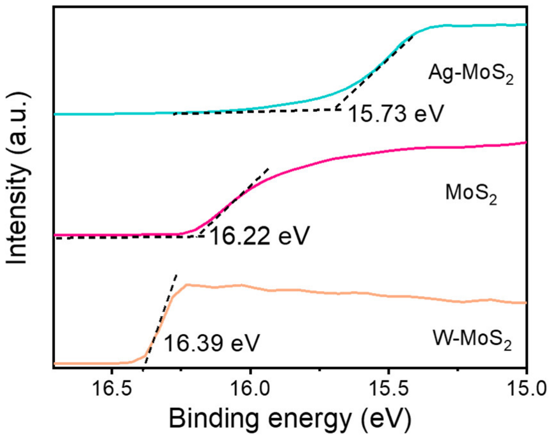

Supplementary Fig. 7 | Work function analysis of catalysts. UPS spectra of Ag-MoS_2, MoS_2, and W-MoS_2 catalysts. Work function = 21.2 eV - $E_{cut\text{-}off}$, 21.2 eV represents the energy of ultraviolet photoelectron.

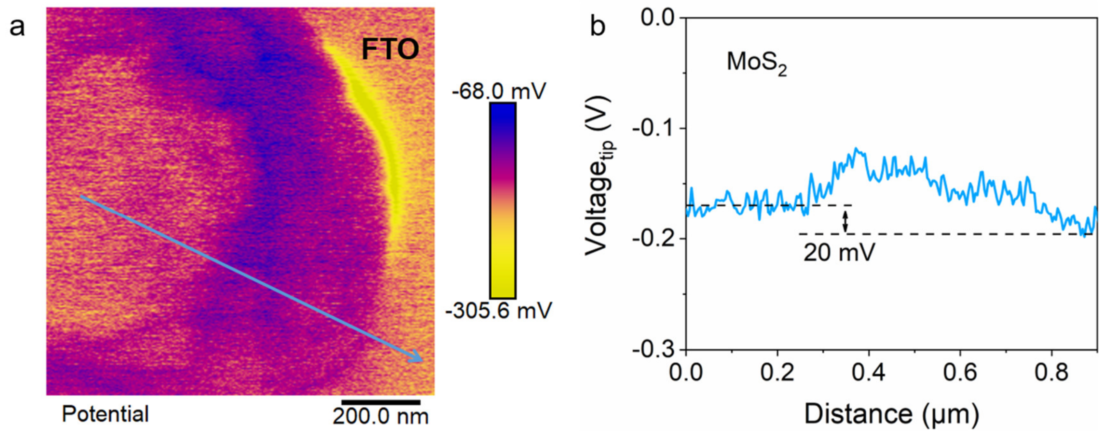

Supplementary Fig. 8 | Work function analysis of catalysts. (a) Surface potential and (b) corresponding line profiles along the arrow lines of MoS_2.

Note: We have tested the KPFM for pure MoS_2 sample. As seen from Supplementary Fig. 8 the surface potential distribution within MoS_2 and FTO substrate region differed, in which the surface potential difference between MoS_2 and FTO substrate was 20 mV. As the work function of FTO was 4.9 eV, the work function of MoS_2 obtained from KPFM technique was 4.92 eV.

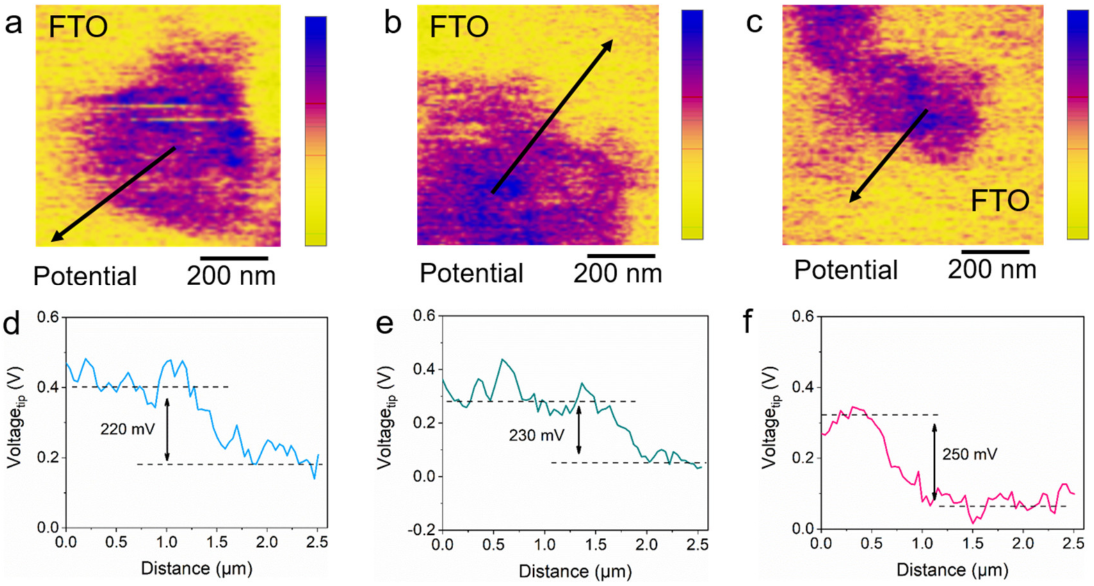

Supplementary Fig. 9 | Work function analysis of catalysts. (a-c) Surface potentials and (d-f) corresponding line profiles along the arrow lines of three branches of Ag-MoS_2 catalysts.

Note: We have also tested the KPFM for three branches of Ag-MoS_2 samples. As seen from Supplementary Fig. 9, the surface potential differences between Ag-MoS_2 and FTO substrate were 220, 230, and 250 mV, respectively. Thus, the work functions of the three branches of Ag-MoS_2 were 5.12 eV, 5.13 eV, and 5.15 eV. The work function difference between Ag-MoS_2 and MoS_2 was around 0.21 eV. Although the work function difference by KPFM test was not that much with UPS and M-S plots, the increased work function can help to verify the positive effect of Ag doping to induce the downward shift of Fermi level position.

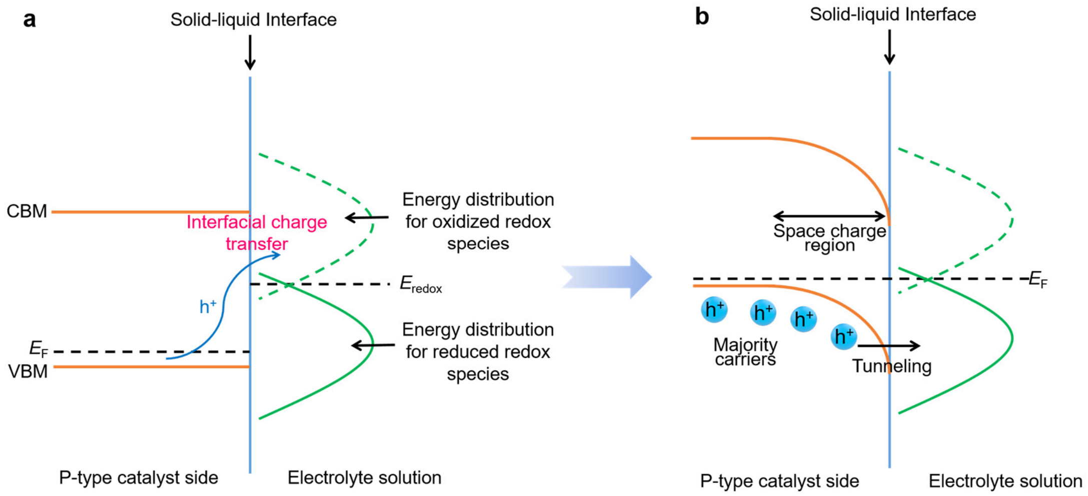

Supplementary Fig. 10 | Interfacial charge transfer between the p-type semiconductor and the electrolyte. The energy level *vs.* position representation of the semiconductor-electrolyte interface for p-type semiconductor electrode (a) before and (b) after contact with the electrolyte solution.

Note: The energy level *vs.* position representation of the semiconductor-electrolyte interface for p-type semiconductor electrode in contact with the electrolyte solution was displayed in Supplementary Fig. 10. For typical p-type catalyst, the Fermi level position was close to the valance band maximum (VBM), which exhibited more positive potential than the redox potential of NO_3^-/NH_3 pair. On the electrolyte solution side, the oxidized redox species (D_{ox}) occupy the region above E_{redox}, while the reduced redox species (D_{red}) occupied the region below E_{redox}. The distribution levels for the D_{red} and D_{ox} in electrolyte were similar to the VB and CB in semiconductors.

As the p-type semiconductor exhibited the characteristic of hole conduction, when the p-type catalyst was immersed in the NO_3^--containing electrolyte, the potential difference between E_F and E_{redox} induced holes (as the majority carriers in p-type semiconductor) transfer from catalyst surface to the solution side. As a result, downward band bending occurred on the semiconductor surface, forming space charge region with hole depletion.

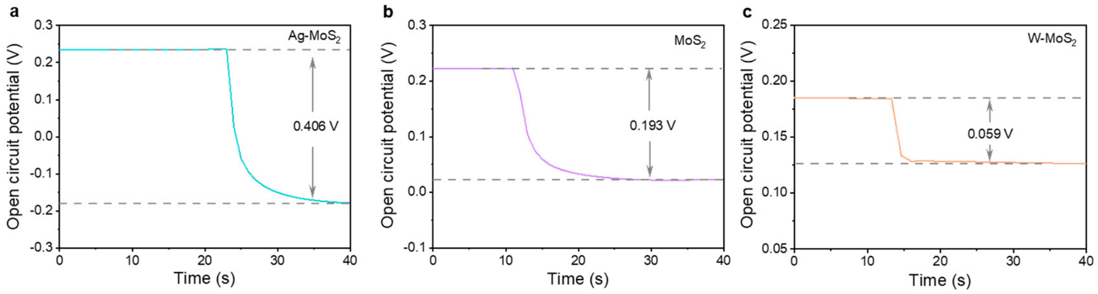

Supplementary Fig. 11 | Surface photovoltage measurements of catalysts. (a) Ag-MoS_2, (b) MoS_2, and (c) W-MoS_2.

Note: The surface photovoltages of the as-prepared samples are obtained by open circuit potential (OCP) measurements under dark and light conditions. Under dark conditions for 10 to 20 s, the OCP values remain constant for all samples. Under illumination, OCP values change obviously and gradually become stable. The surface photovoltages of the samples are the OCP difference between dark and light conditions, which are, 0.406 V for Ag-MoS_2, 0.193 V for MoS_2, and 0.059 V for W-MoS_2 samples.

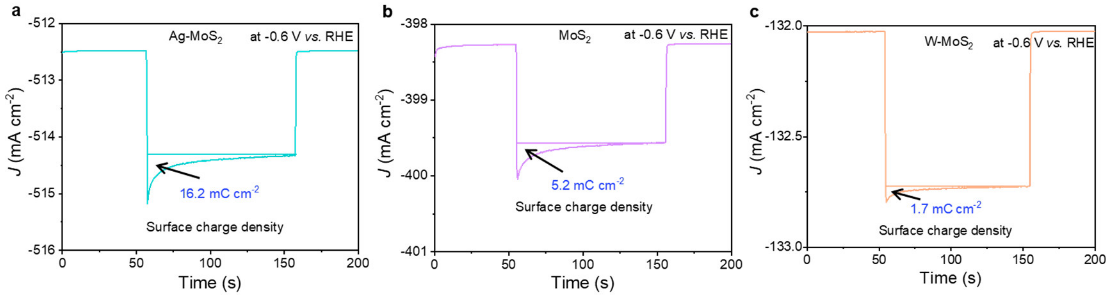

Supplementary Fig. 12 | Transient photocurrent densities of catalysts. (a) Ag-MoS_2, (b) MoS_2, and (c) W-MoS_2 under chopped light conditions at -0.6 V *versus* RHE.

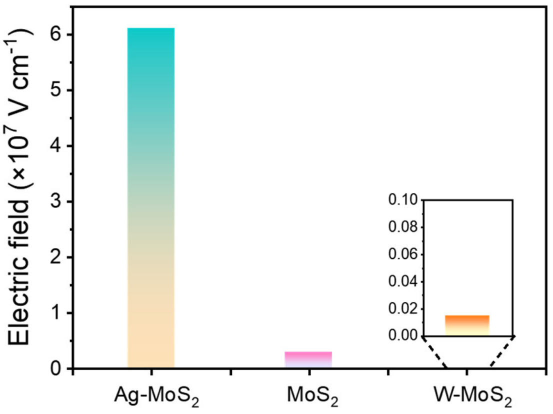

Supplementary Fig. 13 | Built-in electric field strength of catalysts. Calculated electric field intensity within the S-L junction of the as-prepared samples.

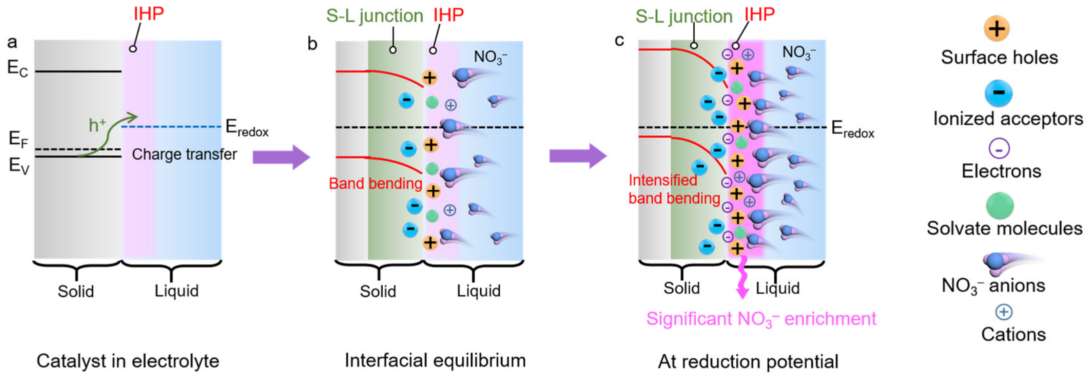

Supplementary Fig. 14 | Solid-liquid (S-L) junction-mediated charge rearrangement to attract NO_3^-. (a) Interfacial thermal equilibrium process when the Ag-MoS_2 contact with the NO_3^- contained solution. Downward band bending at S-L junction under (b) no potential and (c) reduction potential. E_{redox} and E_F represent the theoretical redox potential of NO_3^-/NH_3 and the Fermi level potential. CBM and VBM express the conduction band minimum and valence band maximum of semiconductor, respectively.

Note: For Ag-MoS_2, the Fermi level potential was 1.07 V *versus* RHE, which was much greater than the redox potential of NO_3^-/NH_3 (E_{redox}, 0.27 V *versus* RHE, pH 7). The p-type MoS_2 exhibited the characteristic of hole conduction. When immersing Ag-MoS_2 into the NO_3^--contained solution, the potential difference between the E_F of Ag-MoS_2 and the E_{redox} induced the interfacial charge transfer between semiconductor surface and electrolyte (that is, holes from Ag-MoS_2 surface to electrolyte, Supplementary Fig. 14a). Thus, downward band bending occurred at the solid-liquid (S-L) junction, and the formed positively charged inner Helmholtz plane (IHP) benefited the enrichment of NO_3^- anions (Supplementary Fig. 14b). In addition to the condition without applied potential, the band bending under the working condition (reduction potential) was also investigated (Supplementary Fig. 14c). As the cathode accepted the electrons from the electrochemical workstation, the E_F of Ag-MoS_2 should be closer to the VBM. Therefore, intensified downward band bending should occur at the S-L junction, causing more positively charged IHP region to favor the NO_3^- enrichment at the reduction potential.

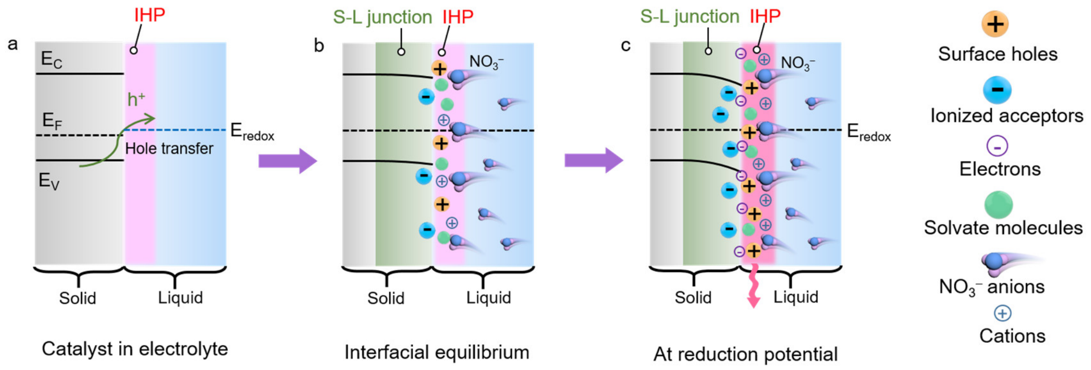

Supplementary Fig. 15 | Solid-liquid (S-L) junction-mediated charge rearrangement to attract NO_3^-. (a) Interfacial thermal equilibrium process when MoS_2 contact with the NO_3^- contained solution. Downward band bending at S-L junction under (b) no potential and (c) reduction potential.

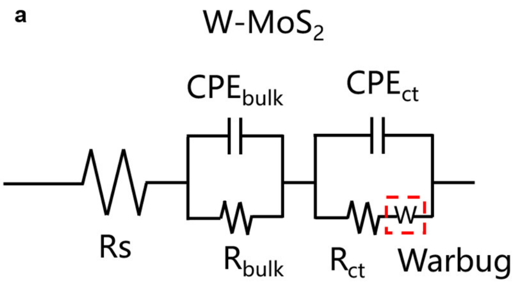

Supplementary Fig. 16 | The fitted equivalent circuit diagram of catalysts. (a) W-MoS_2 and (b) Ag-MoS_2/MoS_2 catalysts at -0.6 V *versus* RHE.

Note: Based on the feature of semicircle at high-frequency and straight line at low-frequency in Fig. 4d, the equivalent circuit of W-MoS_2 was chosen as shown in Supplementary Fig. 16a with a typical Warbug resistance. On the contrary, Ag-MoS_2 and MoS_2 exhibited the two semicircles characteristics at both low- and high-frequency regions. Thus, the equivalent circuit of the two samples was shown in Supplementary Fig. 16b.

In the equivalent circuit diagram, R_s is the external circuit resistance, R_{bulk} is the bulk charge transfer resistance, and R_{ct} is the interfacial charge transfer resistance. In the diagram, R_{bulk} and R_{ct} are paralleled to CPE_{bulk} and CPE_{ct}, respectively. CPE_{bulk} represents the charge accumulation within the bulk catalyst, while CPE_{ct} represents the charge distribution within the solid-liquid interface.

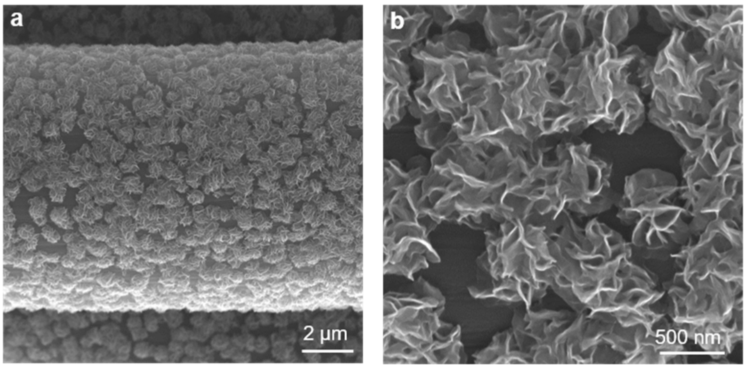

Supplementary Fig. 17 | Morphology characterization of catalyst. SEM images of W-MoS_2 grown on carbon cloth with the scale bars of (a) 2 μm and (b) 500 nm.

.

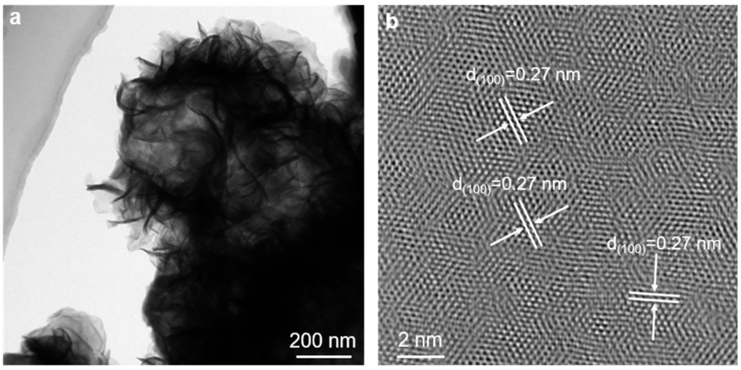

Supplementary Fig. 18 | Structural characterization of catalyst. (a) TEM and (b) HRTEM images of W-MoS_2.

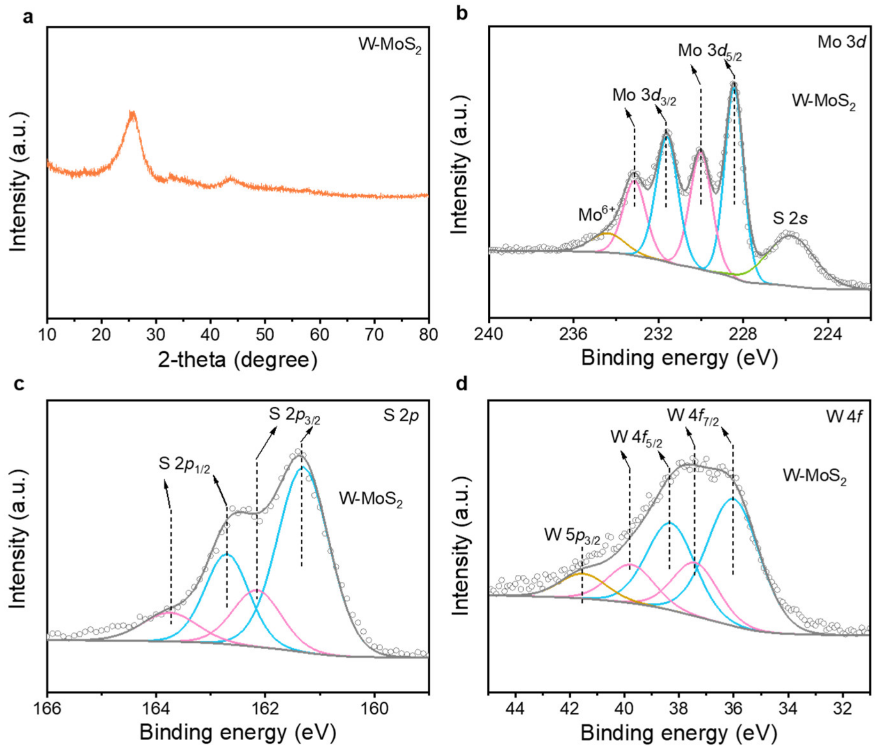

Supplementary Fig. 19 | Structural characterization of catalyst. (a) XRD pattern of W-MoS_2. (b) Mo 3*d*, (c) S 2*p*, and (d) W 4*f* XPS spectra of W-MoS_2.

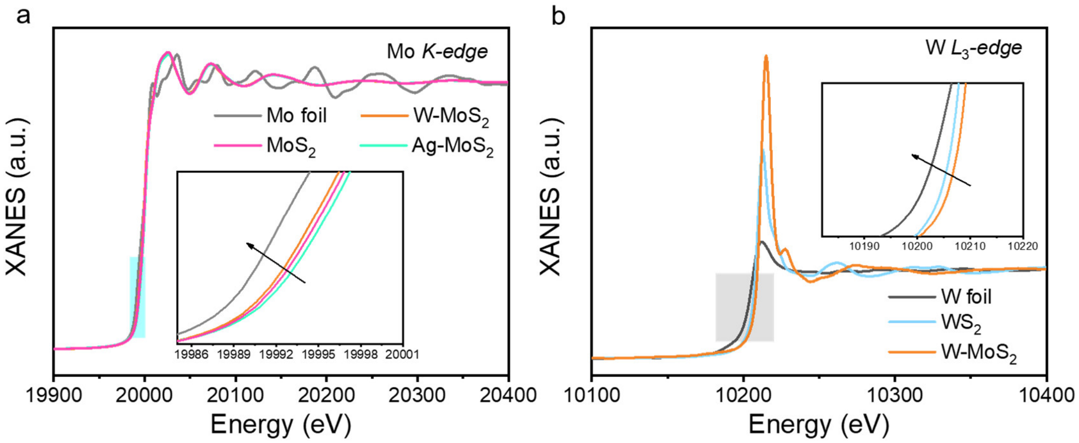

Supplementary Fig. 20 | Electron transfer analysis of catalyst. (a) Mo K-edge XANES spectra, (b) W L-edge XANES spectra of catalysts and reference samples.

Note: As shown in Mo *K*-edge X-ray absorption near edge structure (XANES) spectra (Supplementary Fig. 20a), W-MoS_2 exhibited the higher pre-edge absorption energy than Mo foil, suggesting positively charged Mo atoms. In comparison to MoS_2, the absorption edge of W-MoS_2 underwent a lower energy shift, implying increased electron density on the Mo site upon W incorporation. From the W *L*-edge XANES spectra (Supplementary Fig. 20b), a higher pre-edge absorption edge energy of W-MoS_2 than W foil and WS_2 references reveals the valence state of the W species that exceeds +4. Therefore, there is a significant electronic interaction between the introduced doping species and MoS_2.

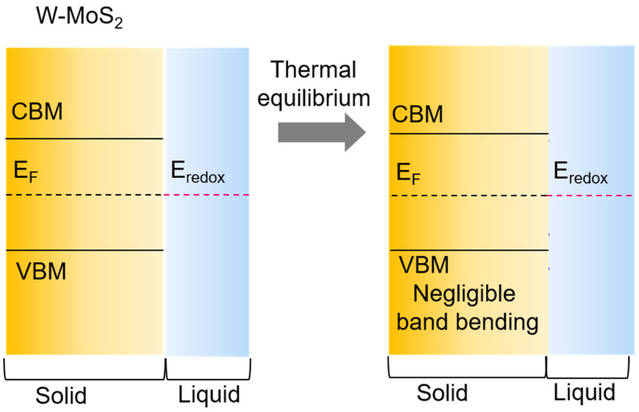

Supplementary Fig. 21 | Schematic diagram for the energy band structure change of catalyst when contacting with NO_3^- electrolyte. W-MoS_2 with comparable E_F to E_{redox} exhibits negligible band bending.

Note: Since the Fermi level potential (E_F) of W-MoS_2 was close to the E_{redox}, negligible hole transfer would occur when immersing W-MoS_2 into NO_3^- electrolyte. Thus, the energy band structure of W-MoS_2 remains intact before and after contacting NO_3^- electrolyte, with no S-L junction on the catalyst surface.

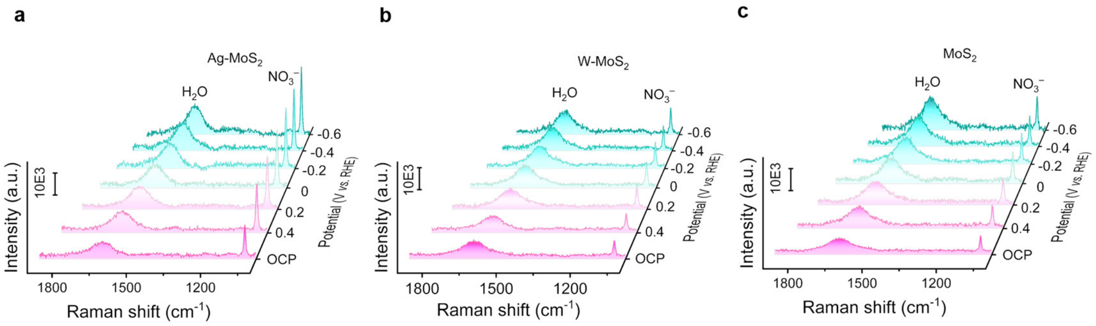

Supplementary Fig. 22 | *In situ* Raman spectra of catalysts. (a) Ag-MoS_2, (b) W-MoS_2 and (c) MoS_2.

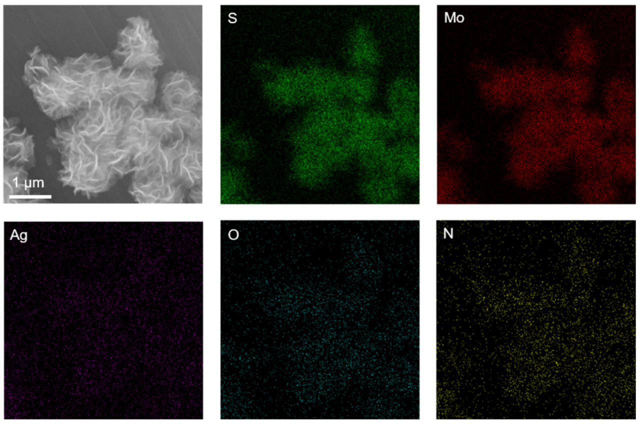

Supplementary Fig. 23 | NO_3^- adsorption capacity analysis of catalysts. Elemental mapping of Ag-MoS_2 at the electrolysis of -0.6 V *versus* RHE in the containing NO_3^- electrolyte.

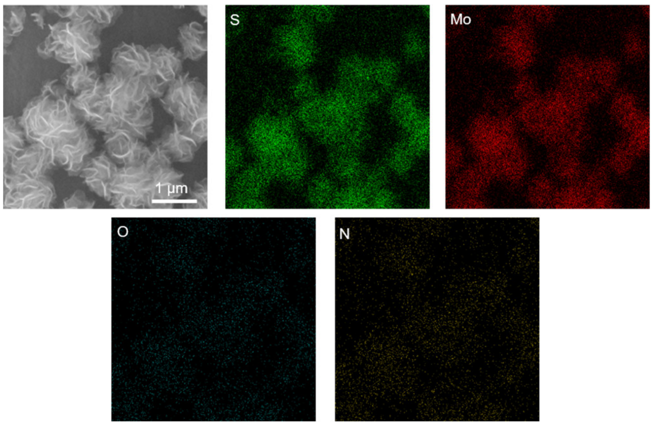

Supplementary Fig. 24 | NO_3^- adsorption capacity analysis of catalysts. Elemental mapping of MoS_2 at the electrolysis of -0.6 V *versus* RHE in the containing NO_3^- electrolyte.

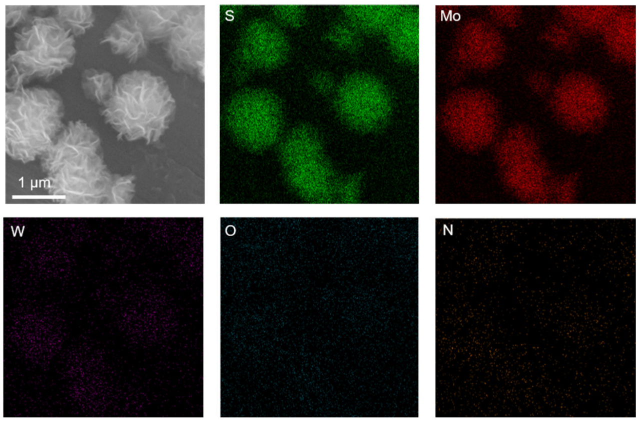

Supplementary Fig. 25 | NO_3^- adsorption capacity analysis of catalysts. Elemental mapping of W-MoS_2 at the electrolysis of -0.6 V *versus* RHE in the containing NO_3^- electrolyte.

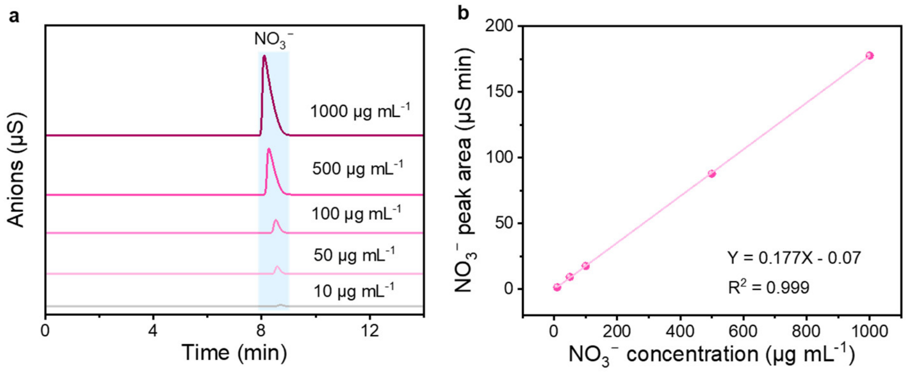

Supplementary Fig. 26 | NO_3^- concentration standard curve. (a) Various concentrations of NO_3^- reference tested by ion chromatography**.** (b) Calibration curve used for calculating the concentration of NO_3^-.

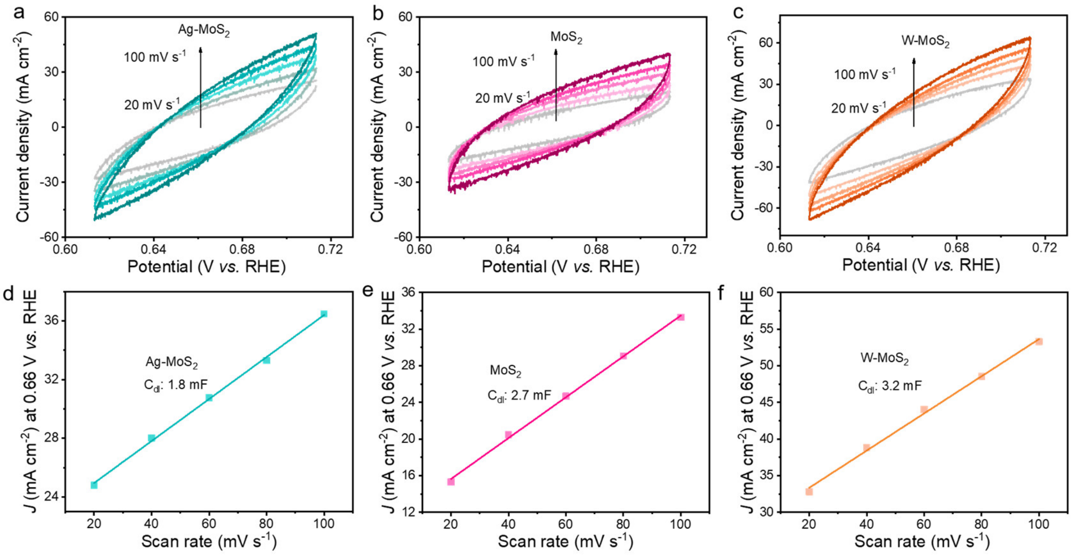

Supplementary Fig. 27 | Capacitance analysis of catalysts. Cyclic voltammetry curves of (a) Ag-MoS_2, (b) MoS_2, (c) W-MoS_2 catalysts at different scan rates, and their (d, e, f) current density differences at 0.66 V *versus* RHE against scan rates to calculate C_{dl}.

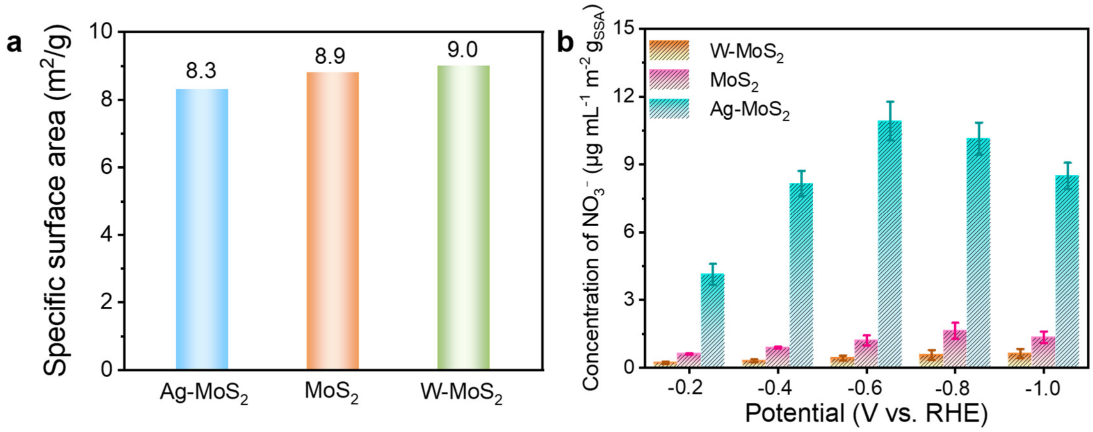

Supplementary Fig. 28 | NO_3^- adsorption capacity analysis of catalysts. (a) Specific surface area of Ag-MoS_2, MoS_2, and W-MoS_2 catalysts tested by nitrogen adsorption/desorption isotherms. (b) The specific surface area (SSA) normalized NO_3^- adsorption capacity at different applied potentials on catalysts.

Note: Nitrogen adsorption/desorption isotherms (Supplementary Fig. 28a) revealed that the specific surface area (SSA) of the catalysts also followed the order: Ag-MoS_2 (8.3 m^2/g) < MoS_2 (8.8 m^2/g) < W-MoS_2 (9.0 m^2/g). When the NO_3^- adsorption capacity was normalized by SSA (Supplementary Fig. 28b), Ag-MoS_2 demonstrated maximum NO_3^- adsorption capacities 5.8 times and 16.0 times higher than those of MoS_2 and W-MoS_2, respectively. This trend aligned with the ECSA normalization results, highlighting Ag-MoS_2's superior intrinsic NO_3^- adsorption capability.

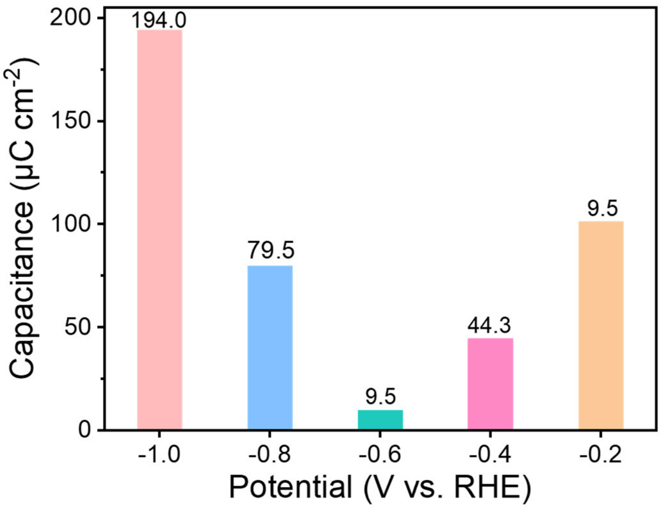

Supplementary Fig. 29 | Capacitance analysis of catalysts. The fitted surface capacitance of Ag-MoS_2 catalyst at different applied potentials from -0.2 to -1.0 V *versus* RHE.

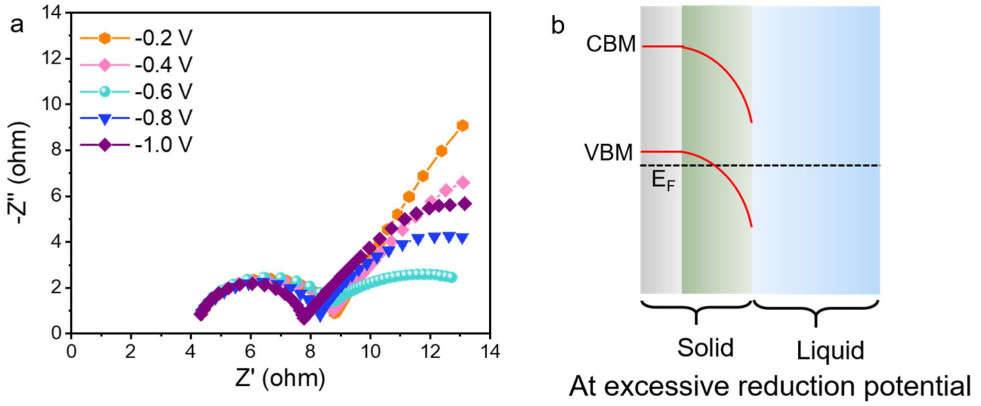

Supplementary Fig. 30 | Formation of degenerate semiconductors under high voltage condition. (a) Nyquist plots of Ag-MoS_2 catalyst at different applied potentials from -0.2 to -1.0 V *versus* RHE. (b) Schematic diagram of the band structure for Ag-MoS_2 at excessive reduction potential (-0.8 and -1.0 V *vs.* RHE), which have become the degenerate semiconductor with metallic property.

Note: Reduction potential can trigger intensified downward band bending within the S-L junction, to boost the built-in electric field and thus the enrichment of NO_3^-. Thus, the NO_3^- concentration increased from -0.2 V to -0.6 V *versus* RHE, in which the Nyquist plot under -0.6 V exhibited the smallest arc radius at the low-frequency region.

However, excessive potential (above -0.6 V) triggered the shift of E_F into the valence band of Ag-MoS_2 (Supplementary Fig. 30b), forming a degenerate semiconductor. In Supplementary Fig. 30a, the decreased arc radius at the high-frequency region suggested promoted conductivity under the potential of -0.8 and -1.0 V, indicating their metallic properties. As a result, the hole transfer from inner catalyst to the surface would be broken, thus hindering the mass transfer of NO_3^- from bulk electrolyte to the IHP. The phenomena had been confirmed by the increased arc radius under the potential of -0.8 and -1.0 V at low-frequency region (Supplementary Table 4). Thus, the NO_3^- concentration peaked under the potential of -0.6 V *versus* RHE.

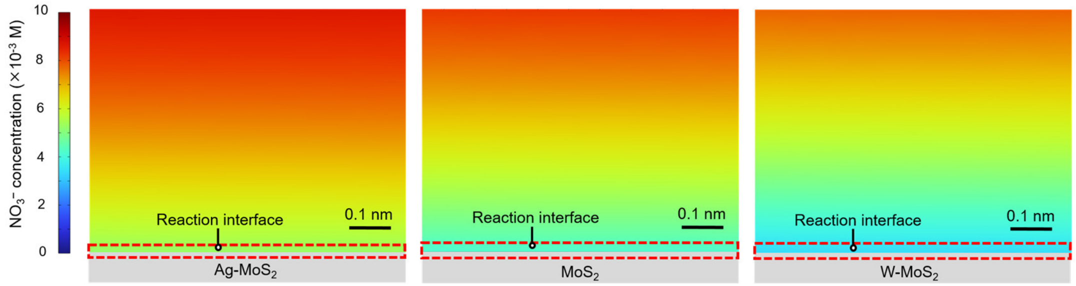

Supplementary Fig. 31 | NO_3^- adsorption capacity analysis of catalysts. The NO_3^- distribution at the solid-liquid interface of Ag-MoS_2, MoS_2, and W-MoS_2 catalysts through COMSOL simulations.

Note: From the simulation results (Supplementary Fig. 31), the concentrations of NO_3^-distributed on the Ag-MoS_2, MoS_2 and W- MoS_2 catalyst surface were 5.1, 4.0, and 3.2 mM, respectively. It was evident that the concentrations of NO_3^- distributed on the Ag-MoS_2 surface was higher than that on its counterpart surfaces, which matched the experimental trends of *in situ* Raman spectroscopy, Bode analysis, elemental mapping, ion chromatography.

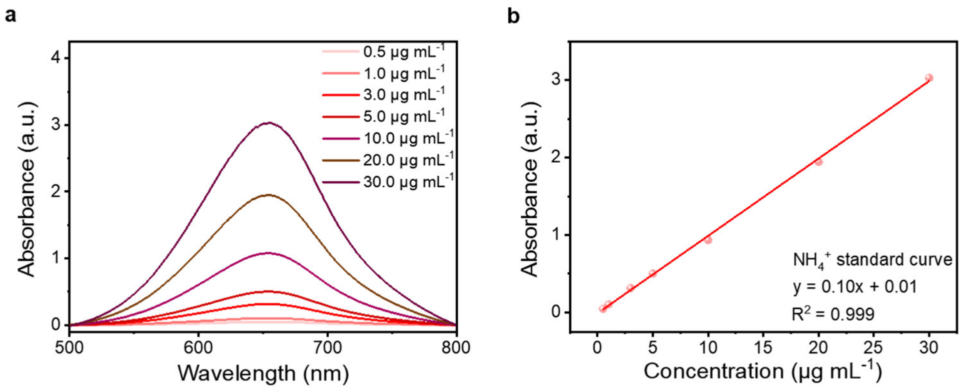

Supplementary Fig. 32 | NH_4^+ concentration standard curve. (a) UV-vis spectra of NH_4^+ reference with various concentrations. (b) The calibration curve of NH_4^+ reference by UV-vis spectra at room temperature.

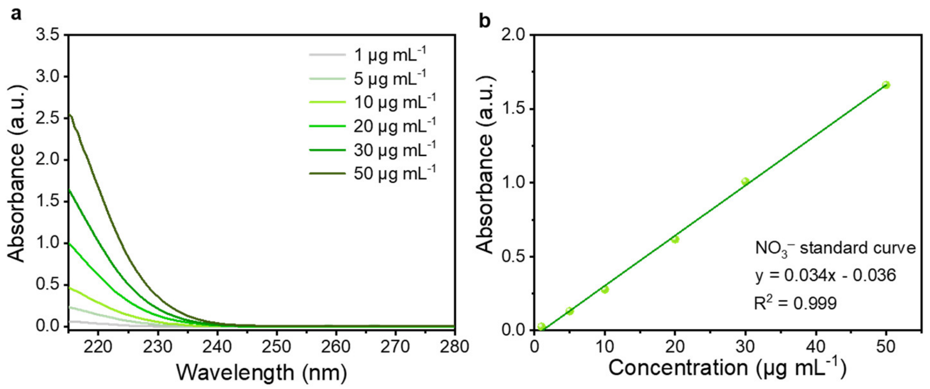

Supplementary Fig. 33 | NO_3^- concentration standard curve. (a) UV-vis spectra of NO_3^- reference with various concentrations. (b) The calibration curve of NO_3^- reference by UV-vis spectra at room temperature.

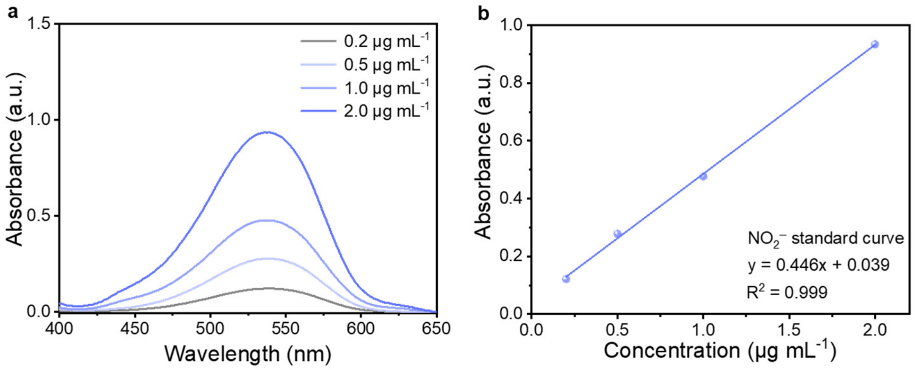

Supplementary Fig. 34 | NO_2^- concentration standard curve. (a) UV-vis spectra of NO_2^- reference with various concentrations. (b) The calibration curve of NO_2^- reference by UV-vis spectra at room temperature.

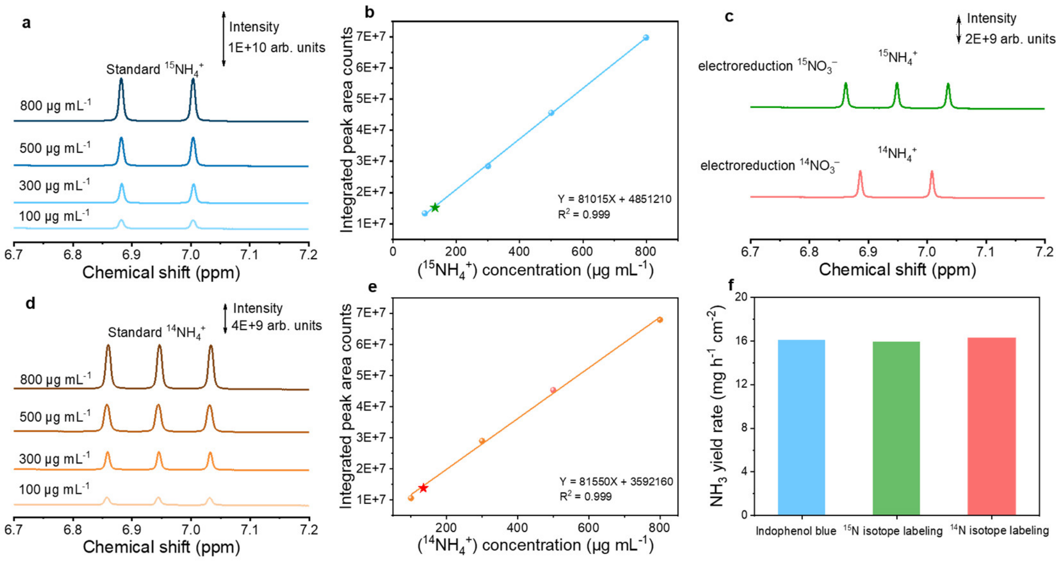

Supplementary Fig. 35 | NH_4^+ concentration standard curve. ^{1}H NMR spectra for (a) $^{15}NH_4Cl$ and (d) $^{14}NH_4Cl$ standard solutions, respectively. Calibration curves for (b) $^{15}NH_4Cl$ and (e) $^{14}NH_4Cl$ standard solutions, respectively. The green star represented the concentrations of produced $^{15}NH_4Cl$ after $^{15}NO_3^-$ electrolysis for 0.5 h. The red star represented the concentrations of produced $^{14}NH_4Cl$ after $^{14}NO_3^-$ electrolysis for 0.5 h. (c) ^{1}H NMR analysis of the electrolyte fed by $^{15}NO_3^-$ and $^{14}NO_3^-$ in NO$_3$RR. (f) The NH_3 yield rate measured by indophenol blue, and isotope labeling method. Reaction conditions: catalyst: Ag-MoS$_2$; potential: -0.6 V *versus* RHE.

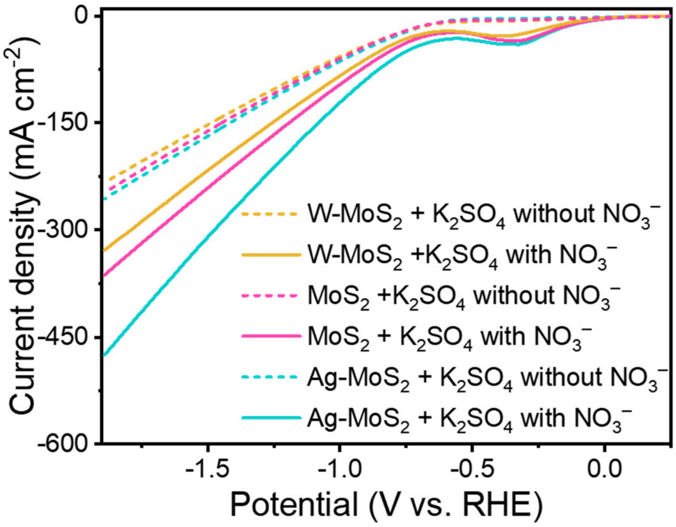

Supplementary Fig. 36 | LSV measurements. LSV curves of the catalysts in electrolytes with and without 1 mM NO_3^- at the scan rate of 1 mV s^{-1}.

Note: To better illustrate the diffusion-limited information NO_3RR, LSV measurements were performed with the NO_3^- concentration of 1 mM and the scan rate of 1 mV s^{-1}. From the applied potential from 0.25 to -1.9 V *vs.* RHE, obvious reduction peaks of all the samples emerges at around -0.27 V vs. RHE, ascribing to the diffusion-limited peak which is the sign of NO_3RR. Compared with counterparts, the highest peak current density of Ag-MoS_2 indicated a fastest surface NO_3RR kinetics, due to the superior NO_3^- enrichment effect.

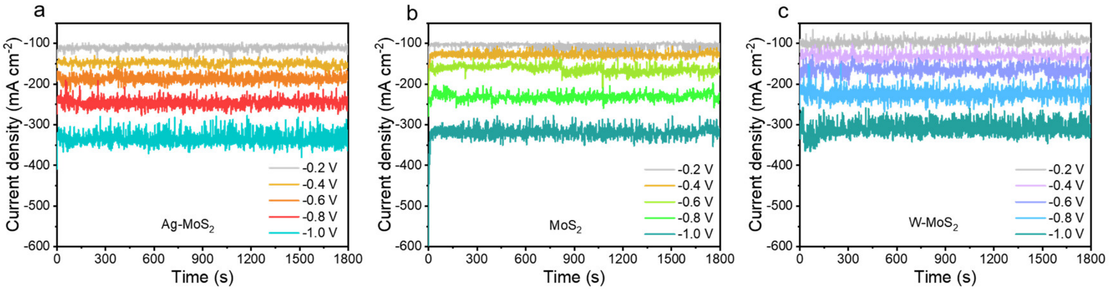

Supplementary Fig. 37 | Time-dependent current density curves of catalysts. (a) Ag-MoS_2, (b) MoS_2, (c) W-MoS_2 at various potentials for 1800 s.

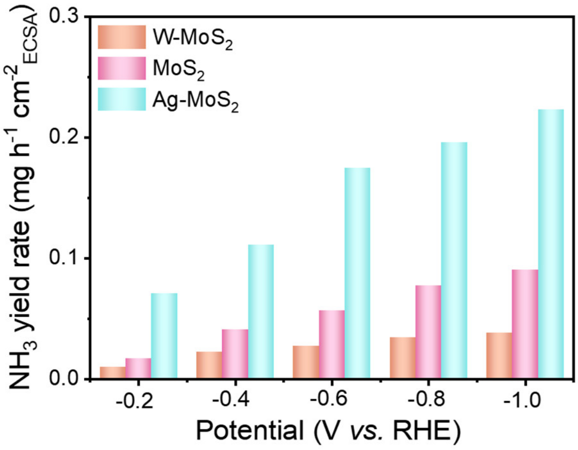

Supplementary Fig. 38 | NO_3RR performance. The electrochemical-surface-area-normalized NH_3 yield rate at different applied potentials on catalysts.

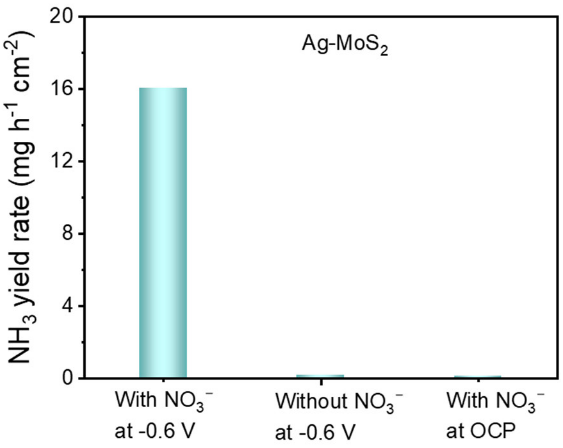

Supplementary Fig. 39 | Performance control experiments. NH_3 yield rate for Ag-MoS_2 under different reaction conditions.

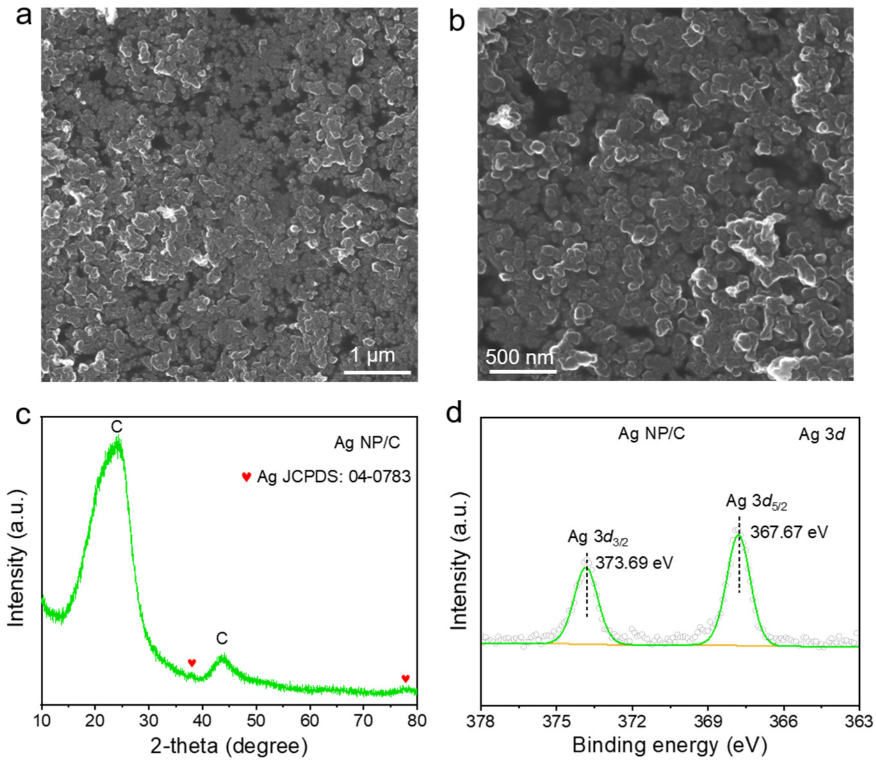

Supplementary Fig. 40 | Structural characterization of comparison sample. (a, b) SEM images, (c) XRD pattern, and (d) XPS spectra of Ag 3*d* orbit in Ag NP/C catalyst.

Note: As seen from Supplementary Figs. 14a-b, the Ag nanoparticles distribute on the carbon black evenly. In the XRD pattern, besides the characteristic peaks of carbon substrate, obvious peaks at 38.1° and 77.4° can be indexed to the (111) and (311) planes of cubic Ag. In high-resolution XPS spectra, the binding energies of 373.69 and 367.67 eV are assigned $3d_{3/2}$ and $3d_{5/2}$ orbits of metallic Ag. These results verified the successful preparation of Ag nanoparticles on carbon black (Ag NP/C).

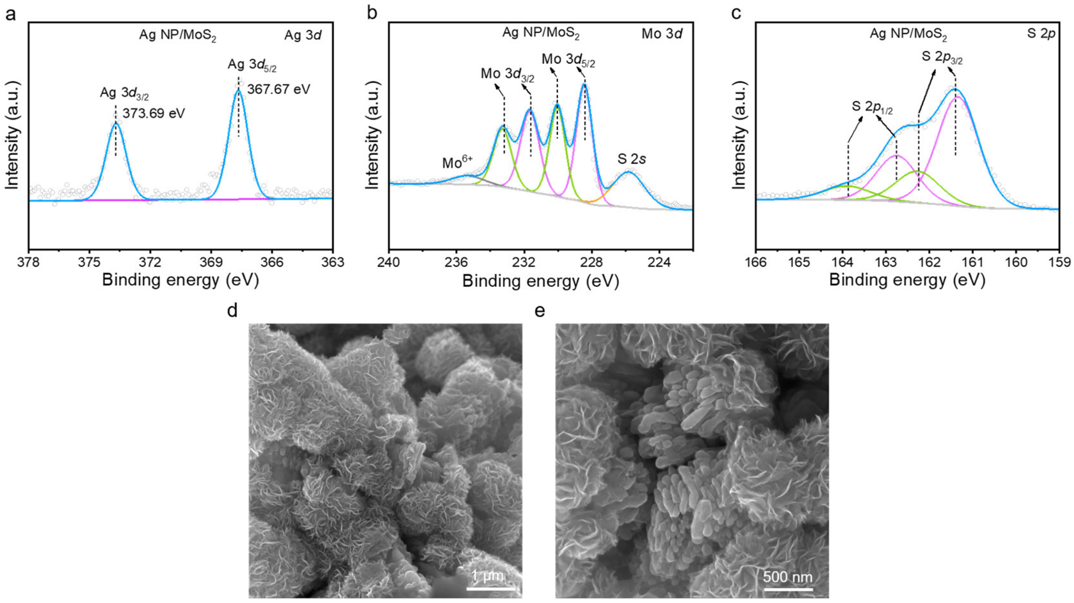

Supplementary Fig. 41 | Structural characterization of comparison sample. XPS spectra of (a) Ag 3*d*, (b) Mo 3*d*, and (c) S 2*p* orbits in Ag NP/MoS_2 catalyst. (d, e) SEM images of Ag NP/MoS_2 sample.

Note: According to the high-resolution XPS spectra, the presence of MoS_2 and metallic Ag can be confirmed. MoS_2 exhibits the nanoflower morphology. After Ag modification, Ag nanoparticles (NP) distribute on the surface of MoS_2, further demonstrating the successful preparation of Ag NP/MoS_2 control samples.

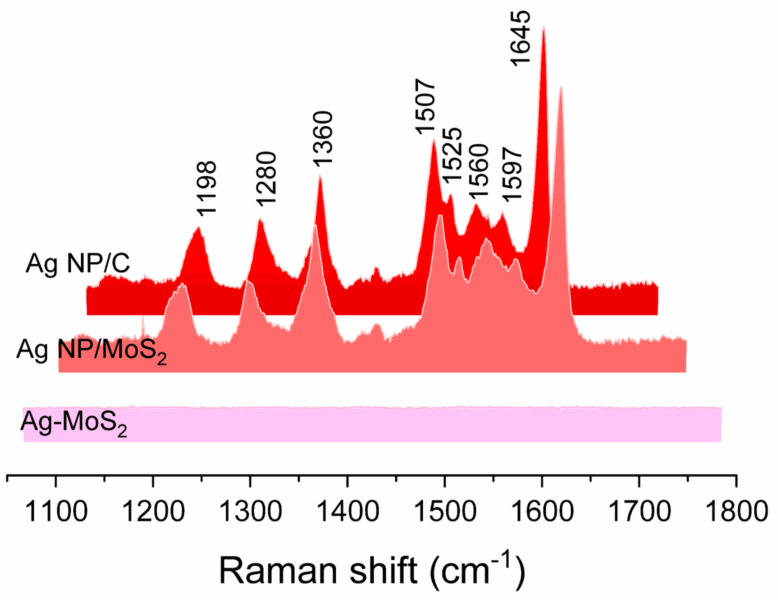

Supplementary Fig. 42 | Eliminating the impact of SERS effect on increased Raman signals. Raman spectra of Ag-MoS_2, Ag NP/MoS_2, and Ag NP/C samples under the treatment of RhB on the surface.

Note: To further eliminate the potential influence of SERS effect on the increased Raman signals on Ag-MoS_2, we treated the Ag-MoS_2, Ag NP/MoS_2, and Ag NP/C catalysts with the RhB solution (concentration of 5 μM), respectively. The Raman test was carried out with the excitation wavelength of 532 nm. As seen from Supplementary Fig. 42, for Ag NP/C and Ag NP/MoS_2 samples, characteristic Raman peaks of RhB emerged. Specifically, the bands center at 1198 and 1525 cm^{-1} are ascribed toaromatic C-H bending in RhB molecule, the peak at 1280 cm^{-1} is the result of C-C bridge-bands stretching, the bands of 1360, 1507, 1560, and 1645 cm^{-1} originate from the aromatic C-C stretching modes, while the peak at 1597 cm^{-1} is attributed to C-H stretching, those are all characteristic vibration modes of RhB dye. The intensity of these RhB characteristic peaks on Ag NP/MoS_2 and Ag NP/C are as high as10^5 level.

While, no obvious Raman peaks are observed of Ag-MoS_2 sample, which further verify that no metallic Ag exists in Ag-MoS_2, and the Ag-MoS_2 sample has no SERS effect. The increased Raman signal of NO_3^- on Ag-MoS_2 can be ascribed to the enrichment effect, which benefits the NO_3RR performance.

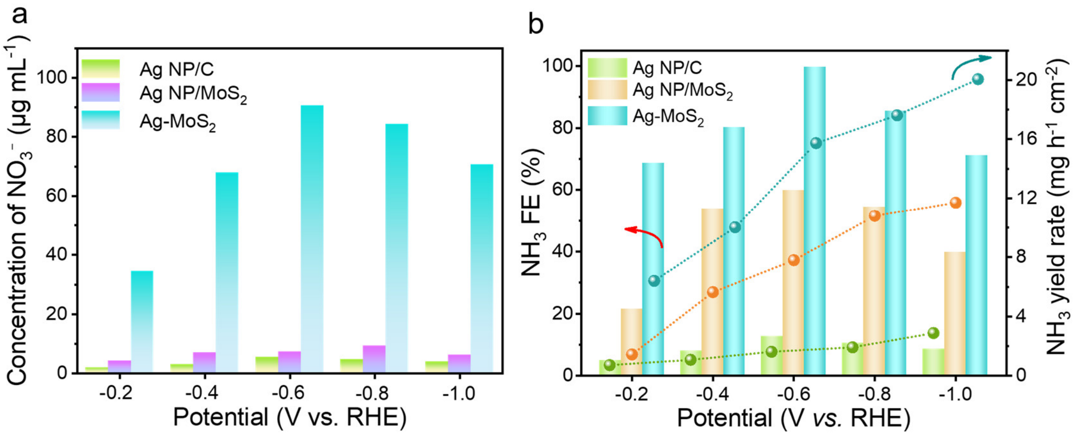

Supplementary Fig. 43 | Controlled experiments. (a) The concentration of NO_3^- adsorbed on Ag NP/C, Ag NP/MoS_2, and Ag-MoS_2 at different applied potentials in NO_3^- containing electrolyte, tested by ion chromatography. (b)NH_3 yield rate and FE of Ag NP/C, Ag NP/MoS_2 and Ag-MoS_2 samples.

Note: We have carried out the NO_3^- adsorption experiment on Ag NP/C, Ag NP/MoS_2 and Ag-MoS_2 samples different applied potentials (Supplementary Fig. 43a). Clearly, the adsorbed NO_3^- concentrations on Ag NP/C and Ag NP/MoS_2 at each potential are inferior to that on Ag-MoS_2 catalyst.

The NH_3 yield rate and FE of the Ag NP/C, Ag NP/MoS_2 and Ag-MoS_2 samples are summarized and displayed in Supplementary Fig. 43b. For Ag NP/C, the FE is as low as 12.5%, with the NH_3 yield of 2.88 mg h^{-1} cm^{-2}, indicating the poor NO_3RR performance of Ag NP/C.

When anchoring the Ag NP on MoS_2 nanoflower (Ag NP/MoS_2), the NH_3 yield rate and FE are 11.7 mg h^{-1} cm^{-2} and 59%, respectively. While, the NO_3RR performance is still much lower than that of Ag-MoS_2 and comparable to MoS_2. Thus, the promoted NO_3RR activity of Ag-MoS_2 can be ascribed to the Ag dopant induced surface NO_3^- enrichment effect.

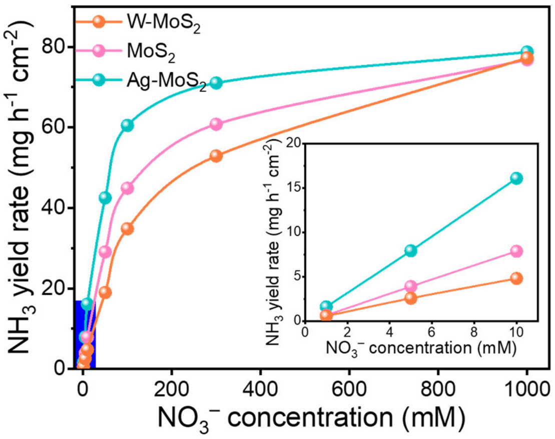

Supplementary Fig. 44 | NO_3RR performance at a wide range of NO_3^- concentrations. NH_3 yield rate of catalysts in different NO_3^- concentrations at -0.6 V *versus* RHE. Insert is the NH_3 yield rate of catalysts in low NO_3^- concentration (≤ 10 mM) from the dark blue region.

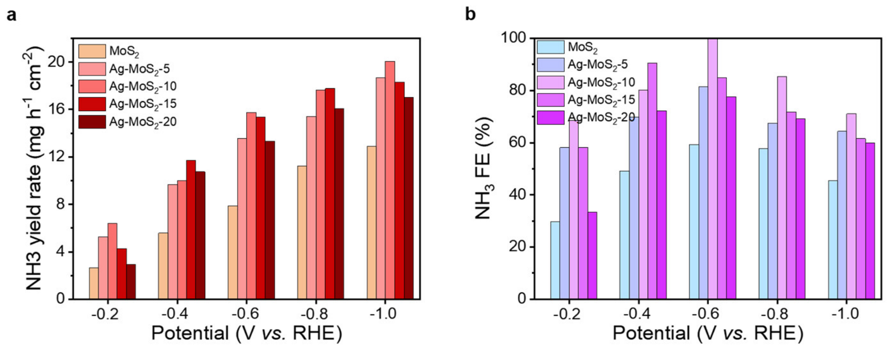

Supplementary Fig. 45 | NO_3RR performance of Ag-MoS_2 with different Ag doping. (a) NH_3 yield rate and (b) NH_3 FE of catalysts in a 0.5 M K_2SO_4 with 10 mM NO_3^- electrolyte in flow cell reactor.

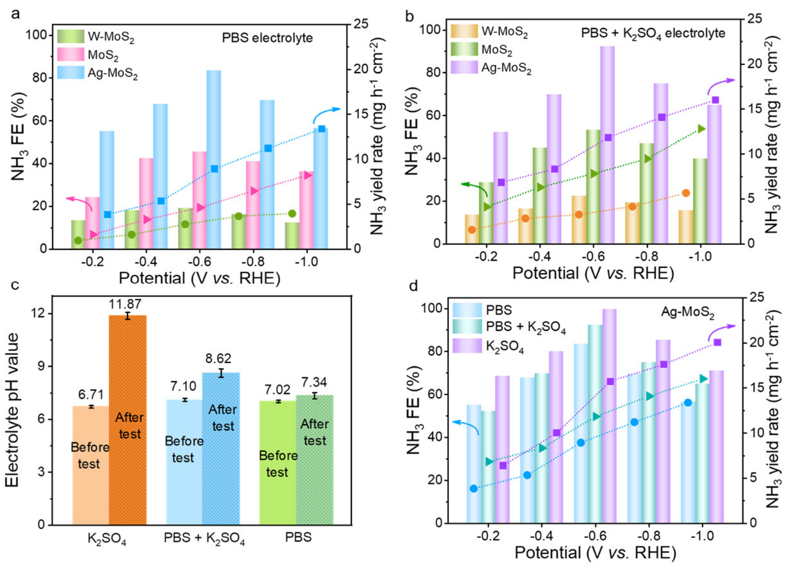

Supplementary Fig. 46 | NO_3RR performance and pH changes of catalysts in different electrolytes. NH_3 FE and NH_3 yield rate of the three samples in (a) PBS electrolyte and (b) PBS + K_2SO_4 hybrid electrolyte. (c) The pH values before and after the NO_3RR process in different electrolytes. (d) NH_3 FE and NH_3 yield rate of Ag-MoS_2 in the three electrolytes.

Note: To probe the NO_3RR performance in buffer solution, we have prepared a 0.5 M PBS solution for all the test (Supplementary Fig. 46a). The NH_3 yield rate of Ag-MoS_2 in PBS solution is 13.4 mg h^{-1} cm^{-2}, with the FE of 83%, which is lower than that in K_2SO_4 electrolyte (Fig. 5a). The NO_3RR performance of MoS_2 and W-MoS_2 in PBS electrolyte are also inferior to that in K_2SO_4 electrolyte. This may be ascribed to the secondary ionization and the relatively low conductivity of PBS. As seen from Supplementary Fig. 46b and Supplementary Fig. 46d, the NH_3 yield rate and FE (16.0 mg h^{-1} cm^{-2}, 92.4%) in the hybrid PBS+K_2SO_4 system was intermediate between those observed in pure PBS (13.4 mg h^{-1} cm^{-2}, 83.4%) and pure K_2SO_4 (20.1 mg h^{-1} cm^{-2}, 99.7%).

Besides, we have measured the pH of both PBS and K_2SO_4 electrolyte before and after NO_3RR. As seen from Supplementary Fig. 46c, the pH of K_2SO_4 electrolyte varied from 6.71 11.87, while the pH of PBS electrolyte changed from 7.02 to 7.08. In addition, the hybrid electrolyte exhibited a pH increase from 7.10 to 8.62, a change more pronounced than in pure PBS (7.02→7.34) but less drastic than in pure K_2SO_4 (6.71→11.87). Within the NO_3RR process, the hydrogenation of N-contained intermediates led to the continuous consumption of H^+. Thus, the neutral K_2SO_4 system changed to alkaline at the end of NO_3RR. The continuous increased pH can not only benefit the inhibition of competitive HER, but also favor the recycle of ammonia products (alkaline environment was conducive to the escape of NH_3).

While, the KH_2PO_4 and K_2HPO_4 are the main components in PBS buffer solution. The secondary dissociation characteristic can counteract the influence of H^+ consumption on the acidity or alkalinity of the solution, thereby maintaining a relatively stable pH value of the solution. Under this condition, the unchanged pH (7.02 to 7.08) may not that effective to inhibit the undesirable HER, leading to the reduction of FE. These results suggested that K_2SO_4 could be available as the supporting electrolyte.

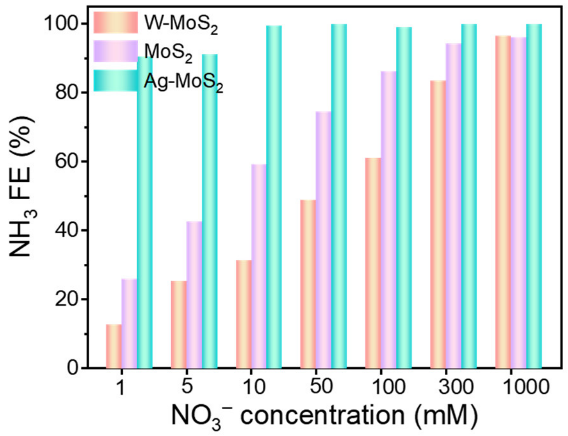

Supplementary Fig. 47 | NH_3 FE at a wide range of NO_3^- concentrations. NH_3 FE of catalysts in a 0.5 M K_2SO_4 electrolyte with different NO_3^- concentrations at -0.6 V *versus* RHE.

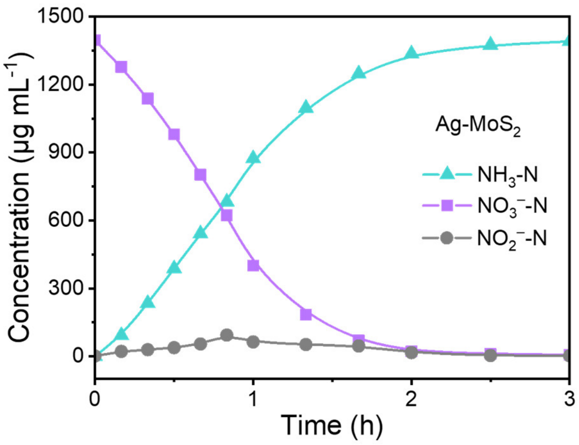

Supplementary Fig. 48 | NO_3^- removal experiments. NO_3^- removal in a 0.5 M K_2SO_4 with 100 mM NO_3^- electrolyte (equals 1400 μg mL^{-1} NO_3^-–N) at -0.6 V *versus* RHE in H-cell reactor. After 3 h electrolysis, only 9.6 μg mL^{-1} of NO_3^-–N and 0.68 μg mL^{-1} of NO_2^-–N remained.

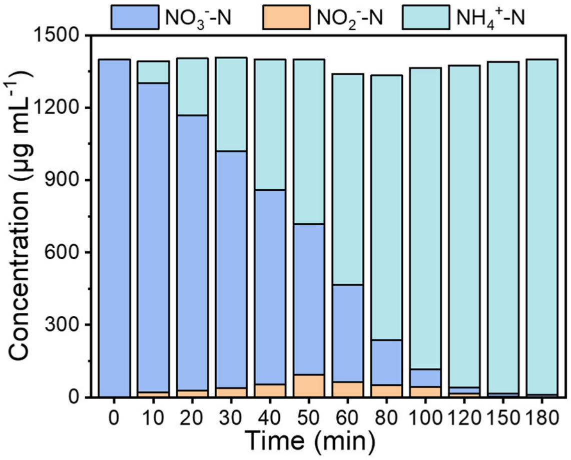

Supplementary Fig. 49 | NO_3^- removal experiments. Product concentrations at different times using Ag-MoS_2 catalysts in 0.5 M K_2SO_4 with 100 mM NO_3^- electrolytes during NO_3RR in H-cell reactor.

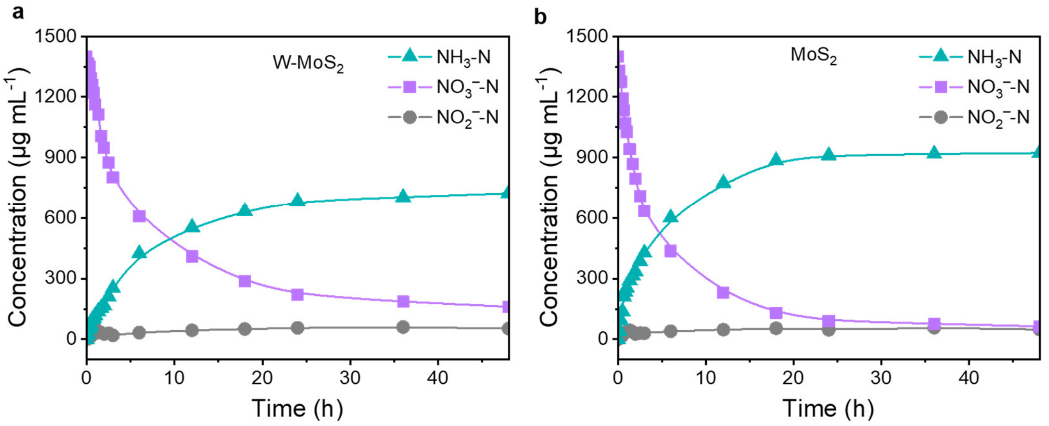

Supplementary Fig. 50 | NO_3^- removal experiments. NO_3^- removal over (a) W-MoS_2 and (b) MoS_2 catalysts at -0.6 V *versus* RHE in H-cell reactor in 0.5 M K_2SO_4 + 100 mM NO_3^- electrolyte (equals 1400 μg mL^{-1} NO_3^-–N).

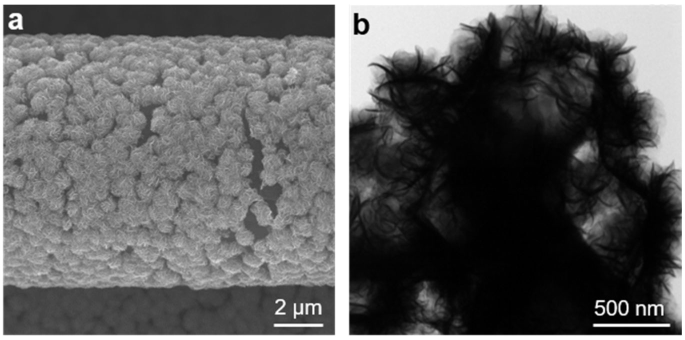

Supplementary Fig. 51 | Morphology characterization after NO_3RR tests. (a) SEM and (b) TEM images of Ag-MoS_2 after NO_3RR.

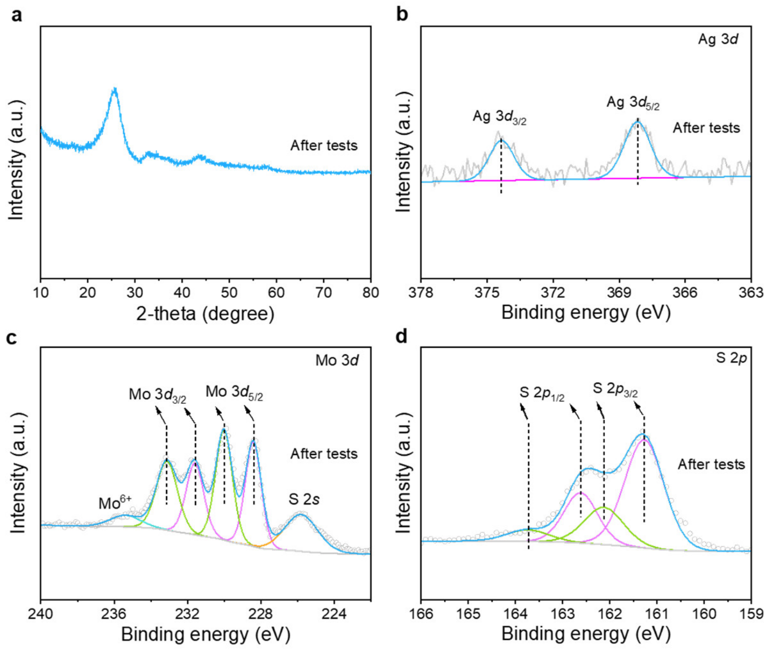

Supplementary Fig. 52 | Structural characterization after NO_3RR tests. (a) XRD patterns, (b) Ag 3*d*, (c) Mo 3*d*, and (d) S 2*p* XPS spectra of Ag-MoS_2 after NO_3RR.

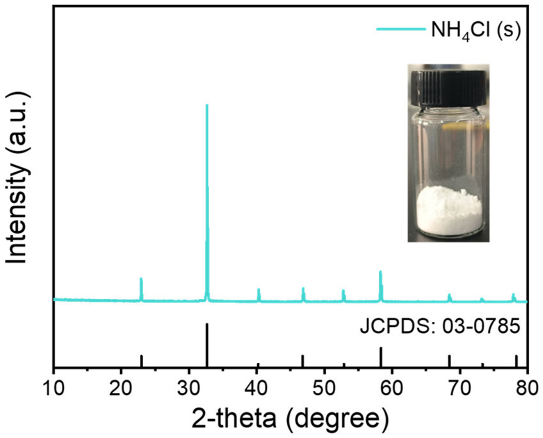

Supplementary Fig. 53 | Ammonia recovery. Synthesized NH_4Cl product and its XRD pattern. Inset: the product itself.

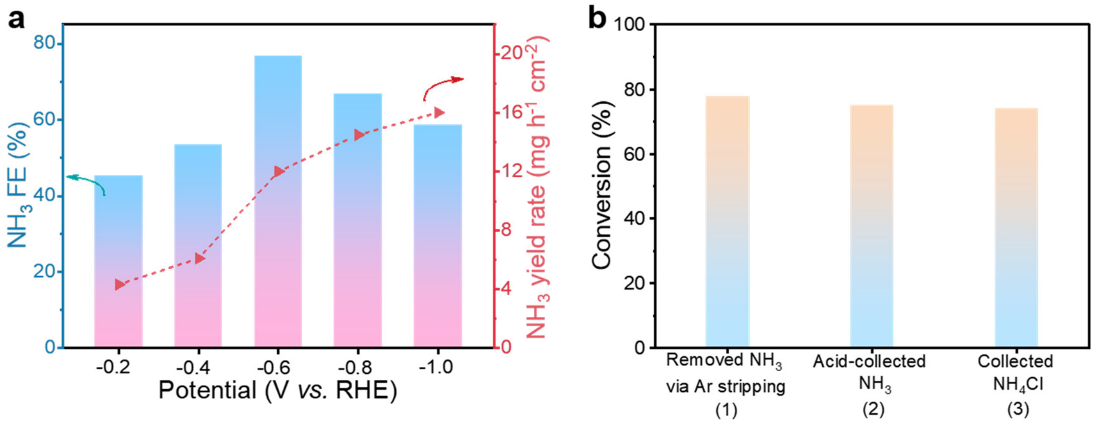

Supplementary Fig. 54 | Wastewater treatment and ammonia recovery capacity of catalysts. (a) NH_3 yield and Faradaic efficiency of Ag-MoS_2 catalyst under different applied potentials in simulated nitrate-containing wastewater. (b) The conversion efficiency of different steps for the ammonia product synthesis process.

Note: We have also utilized the air-stripping method for the recovery of high-purity ammonia products from simulated nitrate-containing wastewater. Clearly, approximately 77.8% of the NH_3 vapor was successfully stripped out from the electrolyte (Supplementary Fig. 54b). Subsequently, around 75.1% of the outflowing NH_3 gas was collected in an HCl solution, and approximately 73.9% of NH_4Cl powder was finally obtained after rotary evaporation. Although the conversion efficiency decreased in complex wastewater environment, this approach still holds the potential for practical application.

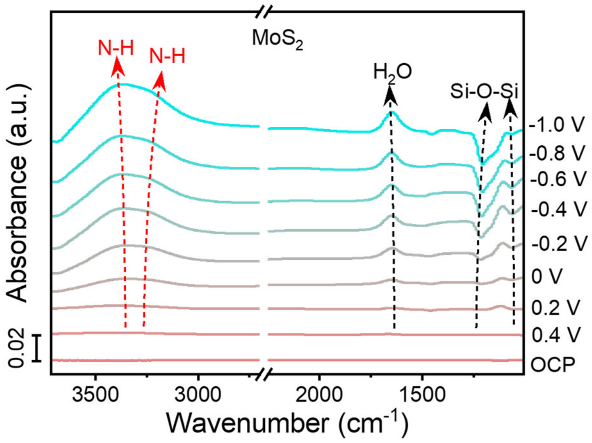

Supplementary Fig. 55 | Mechanistic study on NO_3^- electroreduction. *In situ* ATR-IR spectra of MoS_2 catalysts.

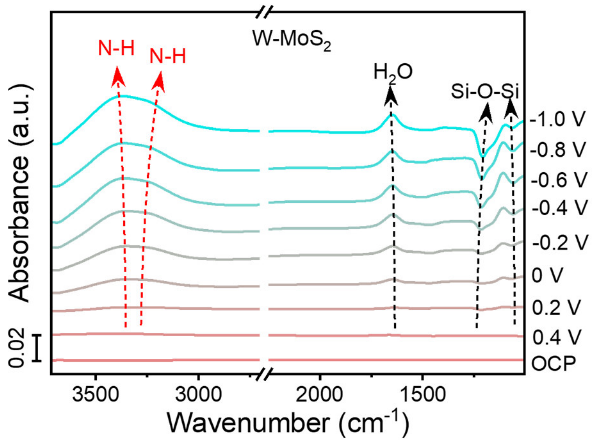

Supplementary Fig. 56 | Mechanistic study on NO_3^- electroreduction. *In situ* ATR-IR spectra of W-MoS_2 catalysts.

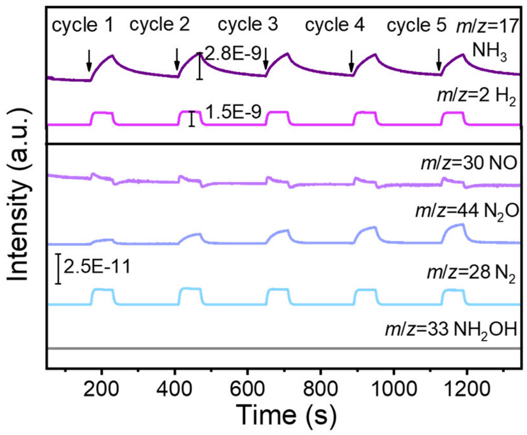

Supplementary Fig. 57 | Molecular intermediates and products analysis during NO_3RR. Online differential electrochemical mass spectrometry (DEMS) measurements of NO_3RR over MoS_2 under the potential of -0.6 V *versus* RHE.

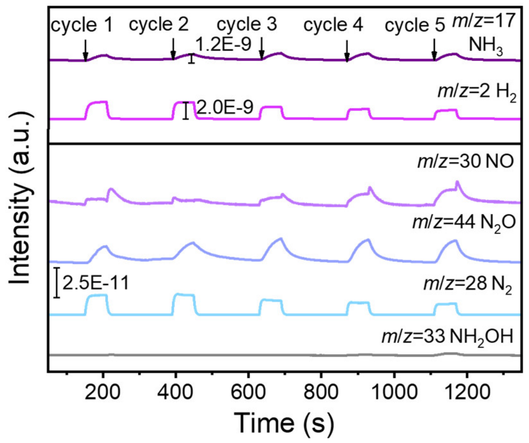

Supplementary Fig. 58 | Molecular intermediates and products analysis during NO_3RR. Online differential electrochemical mass spectrometry (DEMS) measurements of NO_3RR over W-MoS_2 under the potential of -0.6 V *versus* RHE.

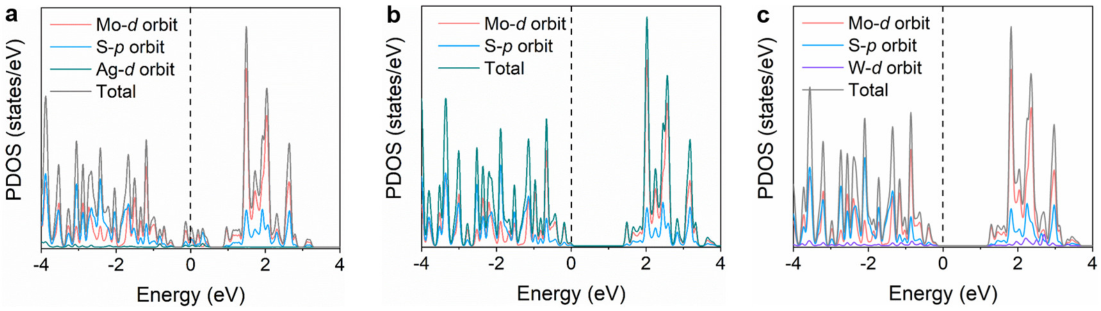

Supplementary Fig. 59 | The partial density of states (PDOS). (a) Ag-MoS_2, (b) MoS_2 and (c) W-MoS_2.

Note: According to the PDOS of the MoS_2-based catalysts, the valance band of MoS_2 was formed by the hybridization of Mo-*d* orbitals and S-*p* orbitals. The Ag doping induced generation of band-tail states near the valance band maximum, which lead to the downward shift of Fermi level. In addition, the W doping triggered the upward shift of Fermi level away from the VBM.

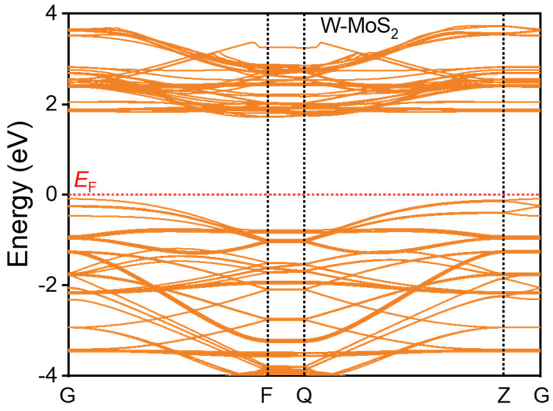

Supplementary Fig. 60 | DFT calculation. Electronic band structures of W-MoS_2.

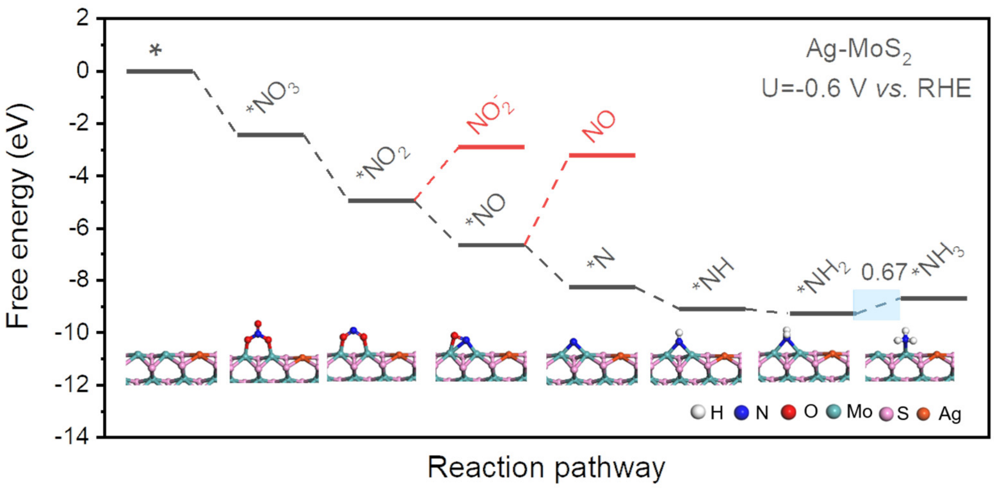

Supplementary Fig. 61 | NO_3RR pathway. Gibbs free energy diagram of various intermediates generated during NO_3RR over Ag-MoS_2 at the potential of -0.6 V *versus* RHE for pH = 7.

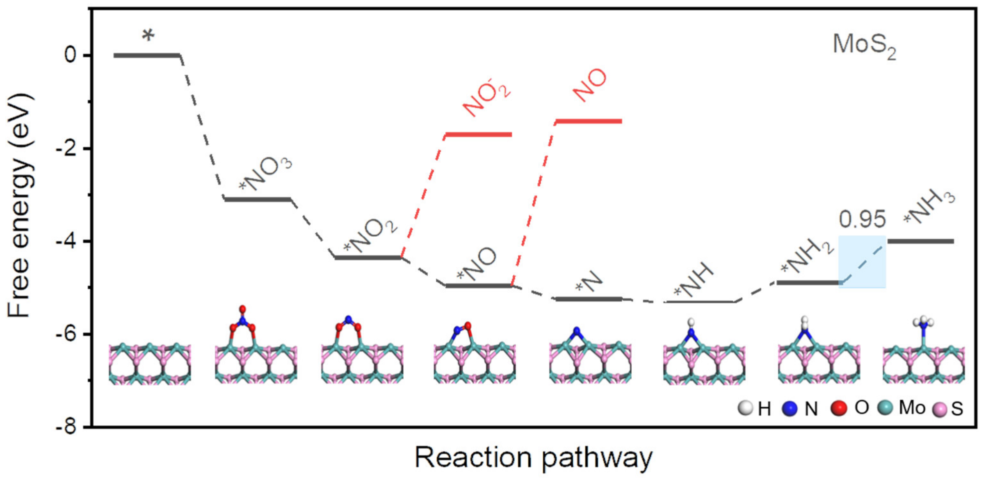

Supplementary Fig. 62 | NO_3RR pathway. Gibbs free energy diagram of various intermediates generated during NO_3RR over MoS_2 at pH = 7.

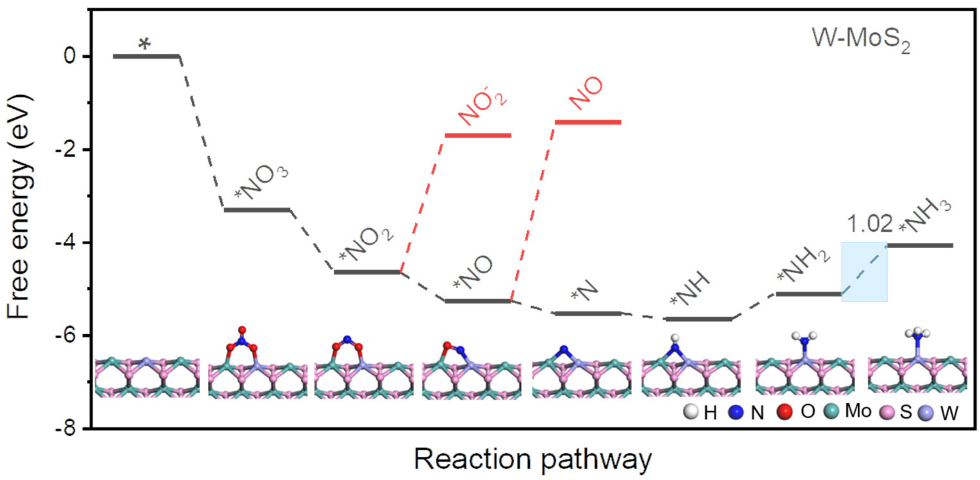

Supplementary Fig. 63 | NO_3RR pathway. Gibbs free energy diagram of various intermediates generated during NO_3RR over W-MoS_2 at pH = 7.

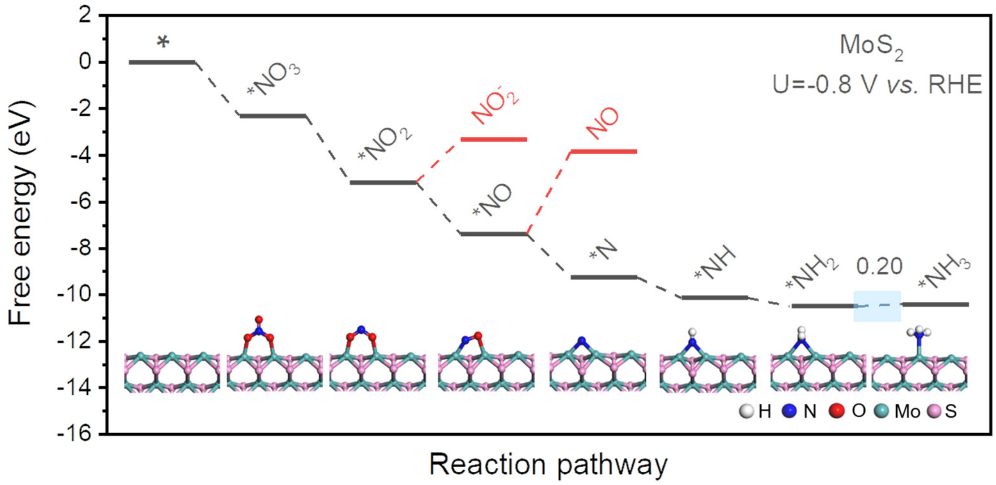

Supplementary Fig. 64 | NO_3RR pathway. Gibbs free energy diagram of various intermediates generated during NO_3RR over MoS_2 at the potential of -0.8 V *vs.* RHE for pH = 7.

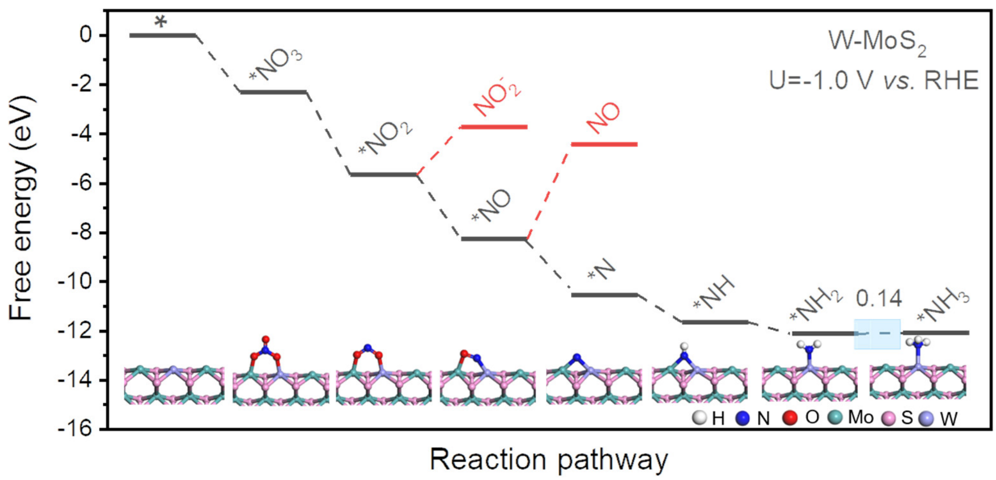

Supplementary Fig. 65 | NO_3RR pathway. Gibbs free energy diagram of various intermediates generated during NO_3RR over W-MoS_2 at the potential of -1.0 V *vs.* RHE for pH = 7.

Supplementary Tables

Supplementary Table 1 | The content of Ag elements for the series Ag-MoS_2 catalysts determined by ICP-OES.

Sample	Ag (wt.%)
Ag-MoS_2-5	1.87
Ag-MoS_2-10	3.49
Ag-MoS_2-15	5.91
Ag-MoS_2-20	6.91

Supplementary Table 2 | Calculated carrier concentration of the three samples by M-S plots.

Samples	Carrier concentration (cm^{-3})
W-MoS_2	3.25×10^{18}
MoS_2	6.3×10^{18}
Ag-MoS_2	1.14×10^{19}

Note: Specifically, the carrier concentrations of the three samples were obtained through Mott-Schottky plots (Fig. 3a). As the slope of Ag-MoS_2 is much lower than those of MoS_2 and W-MoS_2, the calculated carrier concentration of Ag-MoS_2 was 1.14×10^{19} cm^{-3}, 1.8 and 3.5 times of MoS_2 (6.3×10^{18} cm^{-3}) and W-MoS_2 (3.25×10^{18} cm^{-3}). A high carrier concentration can induce intensified surface band bending when the Ag-MoS_2 was contacted with the NO_3^- contained electrolyte.

Supplementary Table 3 | Calculated space charge layer width of the as-prepared samples.

**Samples**	**Space charge layer width (W_{sc})**
MoS_2	10.28 nm
Ag-MoS_2	5.88 nm

Note: To further investigate whether the charge tunneling effect would occur, we have then calculated the SCL width of MoS_2 and Ag-MoS_2. In the case of a p-type semiconductor, the holes were depleted in the SCL of MoS_2 and Ag-MoS_2, and the width is calculated by the equation as follows:

$$W_{sc}=\sqrt{\frac{2\epsilon_r\varepsilon_0(U-U_{FB})}{eN_d}}$$

Where ε_0 is the vacuum permittivity, ϵ_r is the relative dielectric constant of MoS_2, U is the applied external potential, U_{FB} is the flat band potential, and N_d is the donor density.

As a result, the W_{sc} of MoS_2 and Ag-MoS_2 were 10.28 and 5.88 nm, respectively. The Ag doping increased the carrier concentration, and the W_{sc} reduced significantly which is less than 10 nm. Under this condition, the holes had the possibility to tunnel from the semiconductor surface into the solution. Under working condition with applied negative bias, the intensified band bending would further reduce the W_{sc}, to benefit the hole tunneling effect for fast semiconductor-electrolyte interfacial charge transfer.

Supplementary Table 4 | Fitting results for resistances of Ag-MoS_2 catalyst at -0.6 V versus RHE in a 0.5 M K_2SO_4 with 10 mM NO_3^- electrolyte according to EIS equivalent circuit diagram.

Potential (V *vs*. RHE)	R_s (Ω cm^{-2})	R_{bulk} (Ω cm^{-2})	R_{ct} (Ω cm^{-2})
-0.2	4.20	4.78	30.59
-0.4	4.08	4.83	18.73
-0.6	4.18	4.89	6.04
-0.8	4.06	3.69	10.91
-1.0	4.15	3.14	19.35

Supplementary Table 5 | Detailed parameters of COMSOL simulation.

Parameter	Value
D-K^+	2.135×10^{-9} m^2 s^{-1}
D-H^+	9.31×10^{-9} m^2 s^{-1}
D-SO_4^{2-}	1.97×10^{-9} m^2 s^{-1}
D-OH^-	5.273×10^{-9} m^2 s^{-1}
D-NO_3^-	1.902×10^{-9} m^2 s^{-1}
z-K^+	+1
z-H^+	+1
z-NO_3^-	-1
z-OH^-	-1
z-SO_4^{2-}	-2
ε_r	80
F_Constant	96485 C mol^{-1}
C_ NO_3^-	0.01 M

Supplementary Table 6 | Comparison of NO_3RR performance of Ag-MoS_2 with reported works at low nitrate concentration system (≤20 mM).

Catalyst	Electrolyte	NH_3 FE (%)	NH_3 production rate (mg h^{-1} cm^{-2})	Current density (mA cm^{-2})	Ref.
**Ag-MoS_2**	**10 mM KNO_3 + 0.5 M K_2SO_4**	**~100**	**~20**	**~200**	**This work**
$FeNi_{500}$/FF	14.3 mM KNO_3 + 0.05 M Na_2SO_4	65.2	~0.27	~10	11
Co-Fe@Fe_2O_3	500 ppm NO_3^--N + 0.1 M Na_2SO_4	85.2	0.885	~17	12
Cu-PTCDA	8.1 mM KNO_3 + 1 M PBS	85.9	0.44	15	13
Pd	20 mM $NaNO_3$ + 0.1 M NaOH	35	0.34	4.25	14
Cu@C	1 mM KNO_3 + 1 M KOH	72	0.47	5.5	15
CuCI_BEF	7.1 mM KNO_3 + 0.5 M Na_2SO_4	98.6	1.82	62	16
Cu/Cu_2O nanowires	3.2 mM $NaNO_3$ + 0.5 M Na_2SO_4	95.8	4.1	120	17
a-RuO_2	3.2 mM $NaNO_3$ + 0.5 M Na_2SO_4	97.46	2.0	60	18
O-SiNW/Au	10 mM HNO_3 + 0.5 M K_2SO_4	95.6	4.4	_	19

Supplementary Table 7 | Fitting results for resistances of various catalysts at -0.6 V *versus* RHE in a 0.5 M K_2SO_4 with 10 mM NO_3^- electrolyte according to EIS equivalent circuit diagram.

Resistance / Sample tests	R_s (**Ω cm^{-2}**)	R_{bulk} (**Ω cm^{-2}**)	R_{ct} (**Ω cm^{-2}**)	Warburg (**Ω cm^{-2}**)
W-MoS_2	4.21 (0.71%)*	4.80 (0.88%)	47.5 (3.83%)	45.47 (1.96%)
MoS_2	4.25 (0.76%)	4.83 (0.62%)	15.41 (4.25%)	/
Ag-MoS_2	4.18 (0.45%)	4.89 (0.59%)	6.04 (3.17%)	/

* The content in parentheses after each value represents the relative standard deviation of the fitting result.

Note: R_s is the external circuit resistance. R_{bulk} is the bulk trapping resistance. R_{ct} is the interfacial charge transfer resistance. The relative standard deviations (RSD) of the fitted parameters are provided. It is obvious that the RSD of R_s and R_{bulk} values are below 1%, while for R_{ct} and Warburg resistance are below 5%. These further verified the rationality of the selection of the physical equivalent circuit diagram in Supplementary Fig.16.

Supplementary Table 8 | Preparation cost of Ag-MoS_2 on carbon cloth with 1 cm^2.

**Reagents**	**Usage**	**Price (¥)**
$Na_2MoO_4·2H_2O$	0.27 mmol	0.13
CH_4N_2S	1.15 mmol	0.008
$AgNO_3$	0.013 mmol	0.027
$NH_2OH·HCl$	0.65 mmol	$1×10^{-5}$
Hexadecyl trimethyl ammonium bromide	0.011 g	0.01
Carbon cloth	1 cm^2	0.3
**Total**		**0.475**

Note: The present price of industrial grade ammonia and the product NH_4Cl were around 2700~3000 ¥/ton and 1000~1200 ¥/ton, respectively. According to the fabrication process of Ag-MoS_2 catalyst (in experimental section), the preparation cost of Ag-MoS_2 with 1 cm^2 was as low as ca. 0.475 ¥.

Supplementary References

1. Le Formal F., Sivula K., Grätzel M. The transient photocurrent and photovoltage behavior of a hematite photoanode under working conditions and the influence of surface treatments. *J. Phys. Chem. C* **116**, 26707-26720 (2012).
2. Han T. et al. Anion-exchange-mediated internal electric field for boosting photogenerated carrier separation and utilization. *Nat. Commun.* **12**, 4952 (2021).
3. Takashi K.-K., Masayuki M., Hideyuki T., Yoshihiro H., Taneo N. Photoreflectance characterization of built-in potential in MBE-produced as-grown GaAs surface. In: *Proc. SPIE.*) (1990).
4. Ochterski J.W. *Thermochemistry in gaussian*. gaussian inc (2000).
5. Gupta S., Rivera D.J., Shaffer M., Chismar A., Muhich C. Behavior of cupric single atom alloy catalysts for electrochemical nitrate reduction: An Ab initio study. *ACS EST Engg.* **4**, 166–175 (2023).
6. Chen F.Y. et al. Efficient conversion of low-concentration nitrate sources into ammonia on a Ru-dispersed Cu nanowire electrocatalyst. *Nat. Nanotechnol.* **17**, 759-767 (2022).
7. Wu X. et al. Contrasting capability of single atom palladium for thermocatalytic versus electrocatalytic nitrate reduction reaction. *ACS Catal.* **13**, 6804-6812 (2023).
8. Calle-Vallejo F., Huang M., Henry J.B., Koper M.T.M., Bandarenka A.S. Theoretical design and experimental implementation of Ag/Au electrodes for the electrochemical reduction of nitrate. *Phys. Chem. Chem. Phys.* **15**, 3196 (2013).
9. Liu J.-X., Richards D., Singh N., Goldsmith B.R. Activity and selectivity trends in electrocatalytic nitrate reduction on transition metals. *ACS Catal.* **9**, 7052-7064 (2019).
10. Qiao M., Liu J., Wang Y., Li Y., Chen Z. $PdSeO_3$ monolayer: Promising inorganic 2D photocatalyst for direct overall water splitting without using sacrificial reagents and cocatalysts. *J. Am. Chem. Soc.* **140**, 12256-12262 (2018).
11. Wang K. et al. Intentional corrosion-induced reconstruction of defective NiFe layered double hydroxide boosts electrocatalytic nitrate reduction to ammonia. *Nat. Water* **1**, 1068-1078 (2023).
12. Zhang S., Li M., Li J., Song Q., Liu X. High-ammonia selective metal–organic framework–derived co-doped Fe/Fe_2O_3 catalysts for electrochemical nitrate reduction. *PNAS* **119**, e2115504119 (2022).
13. Chen G.-F. et al. Electrochemical reduction of nitrate to ammonia via direct eight-electron transfer using a copper–molecular solid catalyst. *Nat. Energy* **5**, 605-613 (2020).
14. Lim J. et al. Structure sensitivity of Pd facets for enhanced electrochemical nitrate reduction to ammonia. *ACS Catal.* **11**, 7568-7577 (2021).
15. Song Z., Liu Y., Zhong Y., Guo Q., Zeng J., Geng Z. Efficient electroreduction of nitrate into ammonia at ultralow concentrations via an enrichment effect. *Adv. Mater.* **34**, e2204306 (2022).
16. Sun W.J. et al. Built-in electric field triggered interfacial accumulation effect for efficient nitrate removal at ultra-low concentration and electroreduction to ammonia. *Angew. Chem. lnt. Ed.* **60**, 22933-22939 (2021).
17. Wang Y., Zhou W., Jia R., Yu Y., Zhang B. Unveiling the activity origin of a copper-

based electrocatalyst for selective nitrate reduction to ammonia. *Angew. Chem. lnt. Ed.* **59**, 5350-5354 (2020).

18. Wang Y., Li H., Zhou W., Zhang X., Zhang B., Yu Y. Structurally disordered RuO_2 nanosheets with rich oxygen vacancies for enhanced nitrate electroreduction to ammonia. *Angew. Chem. lnt. Ed.* **61**, e202202604 (2022).
19. Kim H.E., Kim J., Ra E.C., Zhang H., Jang Y.J., Lee J.S. Photoelectrochemical nitrate reduction to ammonia on ordered silicon nanowire array photocathodes. *Angewandte Chemie* **134**, e202204117 (2022).
