# Supplementary material for: Near-Unity Nitrate to Ammonia conversion via reactant enrichment at the solid-liquid interface
Source: Nat Commun. 2025 Jul 1;16:5715. doi: 10.1038/s41467-025-60671-y (PMC12219233; doi:10.1038/s41467-025-60671-y)
Supplement: Supplementary file 2 — Description of Additional Supplementary Files [file 41467_2025_60671_MOESM2_ESM.pdf]

**Description of Additional Supplementary Files**

File Name: Supplementary Data 1
Description: Atomic coordinates of DFT calculation
